# Supplementary material for: Bounds for survival probabilities in supercritical Galton-Watson processes and applications to population genetics
Source: J Math Biol. 2026 Feb 26;92(3):40. doi: 10.1007/s00285-026-02349-7 (PMC12945936; doi:10.1007/s00285-026-02349-7)

Supplementary Mathematica notebook:

Bounds for survival probabilities in supercritical Galton-Watson Processes and applications to population genetics

Reinhard Bürger

1. Important definitions

1.1. Generating functions

The notation is essentially the same as in the ms

In[∘]:=
```
φFL[p_, r_][x_] := (r + x (1 - p - r))/(1 - p x);
φPoi[m_][x_] := ⅇ^(-m (1-x));
φBin[n_, p_][x_] := (1 - p + p x)^n;
φNB[r_, p_][x_] := p^r/(1 - (1 - p) x)^r;
φF3[p0_, p2_, p3_][x_] := p0 + (1 - p0 - p2 - p3) x + p2 x^2 + p3 x^3;
```

In[∘]:=
```
φGP[μ_, 0.][x_] := ⅇ^(-μ (1-x));
φGP[μ_, 0][x_] := ⅇ^(-μ (1-x));
φGP[μ_, λ_][x_] := Exp[-μ (1 + ProductLog[-x λ Exp[-λ]]/λ)]
```

In some publications the generating function φGP is incorrect (essentially, with μ and λ swapped); it is correct, for instance, in the book by Johnson and Kotz.

1.2. Definitions of ultimate extinction and survival probabilities, of gamma = φ'(Pinf), and of mean and variance

In[◦]:=
```
PinfFL[p_, r_] := r/p;
gammaFL[p_, r_] := (1 - p)/(1 - r);
meanFL[p_, r_] := (1 - r)/(1 - p);
varFL[p_, r_] := ((1 - r) (p + r))/((1 - p)^2)
```

In[◦]:=
```
Simplify[Solve[φPoi[m][x] == x, x]]
```

Solve: Inverse functions are being used by Solve, so some solutions may not be found; use Reduce for complete solution information.

Out[◦]=
```
{{x → -ProductLog[-e^-m m]/m}}
```

In[◦]:=
```
PinfPoi[m_] := -ProductLog[-e^-m m]/m;
gammaPoi[m_] := -ProductLog[-e^-m m];
SinfPoi[m_] := 1 - PinfPoi[m];
```

In[◦]:=
```
PinfF3[p0_, p2_, p3_] := (Sqrt[4 p0 p3 + (p2 + p3)^2] - (p2 + p3))/(2 p3);

gammaF3[p0_, p2_, p3_] := 1 - ((p2 + 3 p3) Sqrt[4 p0 p3 + (p2 + p3)^2] - 4 p0 p3 - (p2 + p3)^2)/(2 p3);

meanF3[p0_, p2_, p3_] := 1 - p0 + p2 + 2 p3;
varF3[p0_, p2_, p3_] := -p0^2 + p2 + 4 p3 - (p2 + 2 p3)^2 + p0 (1 + 2 p2 + 4 p3);
```

In the following cases, Pinf needs to be calculated numerically :

In[◦]:=
```
PinfBin[n_, p_] := x /. FindRoot[φBin[n, p][x] == x, {x, 0.5}];
gammaBin[n_, p_] := (n p PinfBin[n, p])/(1 - p + p PinfBin[n, p]);
meanBin[n_, p_] := n p;
varBin[n_, p_] := n p (1 - p);
SinfBin[n_, p_] := 1 - PinfBin[n, p]
```

In[∘]:=
```
PinfNB[r_, p_] := x /. FindRoot[φNB[r, p][x] == x, {x, 0.5}];
gammaNB[r_, p_] := -r (-1 + p) p^r (1 + (-1 + p) PinfNB[r, p])^(-1-r);
meanNB[r_, p_] := r (1 - p)/p;
varNB[r_, p_] := r (1 - p)/(p^2);
SinfNB[r_, p_] := 1 - PinfNB[r, p]
```

In[∘]:=
```
PinfGP[μ_, λ_] := x /. FindRoot[φGP[μ, λ][x] == x, {x, 0.5}];
gammaGP[μ_, 0] := gammaPoi[μ];
gammaGP[μ_, λ_] := -(e^(-μ - μ ProductLog[-e^-λ PinfGP[μ,λ] λ]/λ) μ ProductLog[-e^-λ PinfGP[μ, λ] λ]) /
    (PinfGP[μ, λ] λ (1 + ProductLog[-e^-λ PinfGP[μ, λ] λ]));
meanGP[μ_, λ_] := μ/(1 - λ);
varGP[μ_, λ_] := μ/((1 - λ)^3);
SinfGP[μ_, λ_] := 1 - PinfGP[μ, λ];
```

1.3. The (prospective) bounding fractional linear generating functions

First determine the parameters π and ρ:

In[∘]:=
```
Simplify[Solve[PinfFL[p, r] == PinfPoi[m] && gammaFL[p, r] == gammaPoi[m], {p, r}]]
```

Out[∘]=

$$\left\{\left\{p \to \frac{m\,(1+\text{ProductLog}[-e^{-m}\,m])}{m-\text{ProductLog}[-e^{-m}\,m]^2},\ r \to \frac{\text{ProductLog}[-e^{-m}\,m]\,(1+\text{ProductLog}[-e^{-m}\,m])}{-m+\text{ProductLog}[-e^{-m}\,m]^2}\right\}\right\}$$

In[∘]:=
```
substprPoi[m_] :=
  {p → m (1 + ProductLog[-e^-m m])/(m - ProductLog[-e^-m m]^2), r → -ProductLog[-e^-m m] (1 + ProductLog[-e^-m m])/(m - ProductLog[-e^-m m]^2)};
```

In[∘]:=
```
FullSimplify[Flatten[Solve[φFL[p, r][PinfF3[p0, p2, p3]] == PinfF3[p0, p2, p3] &&
    (D[φFL[p, r][x], x] /. x → PinfF3[p0, p2, p3]) == gammaF3[p0, p2, p3], {p, r}]]]
```

Solve: There may be values of the parameters for which some or all solutions are not valid.

Out[∘]=

$$\left\{p \to \frac{1}{-\frac{1}{2}+\frac{1}{\sqrt{4\,p0\,p3+(p2+p3)^2}}+\frac{-p2+\sqrt{4\,p0\,p3+(p2+p3)^2}}{2\,p3}},\ r \to \frac{2\,p0\,\sqrt{4\,p0\,p3+(p2+p3)^2}}{p2+p3+(1+2\,p0)\,\sqrt{4\,p0\,p3+(p2+p3)^2}}\right\}$$

In[∘]:=
```
substprF3[p0_, p2_, p3_] :=
  {p → 2 p3 Sqrt[4 p0 p3 + (p2 + p3)^2]/(2 (1 + 2 p0) p3 - (p2 + p3) (-p2 - p3 + Sqrt[4 p0 p3 + (p2 + p3)^2])),
   r → 2 p0 Sqrt[4 p0 p3 + (p2 + p3)^2]/(p2 + p3 + (1 + 2 p0) Sqrt[4 p0 p3 + (p2 + p3)^2])};
```

The following are computed numerically:

```
In[•]:= substprBin[nBin_, pBin_] :=
         Flatten[NSolve[φFL[p, r][PinfBin[nBin, pBin]] == PinfBin[nBin, pBin] &&
            (D[φFL[p, r][x], x] /. x → PinfBin[nBin, pBin]) == gammaBin[nBin, pBin], {p, r}]]
```

```
In[•]:= substprNB[rNB_, pNB_] :=
         Flatten[NSolve[φFL[p, r][PinfNB[rNB, pNB]] == PinfNB[rNB, pNB] &&
            (D[φFL[p, r][x], x] /. x → PinfNB[rNB, pNB]) == gammaNB[rNB, pNB], {p, r}]]
```

```
In[•]:= substprGP[μ_, λ_] := Flatten[NSolve[φFL[p, r][PinfGP[μ, λ]] == PinfGP[μ, λ] &&
            (D[φFL[p, r][x], x] /. x → PinfGP[μ, λ]) == gammaGP[μ, λ], {p, r}]]
```

Here are fractional linear generating functions that approximate the given one :

```
In[•]:= φPoiFL[m_][x_] := (φFL[p, r] /. substprPoi[m])[x];
        φBinFL[n_, pp_][x_] := (φFL[p, r] /. substprBin[n, pp])[x];
        φNBFL[rr_, pp_][x_] := (φFL[p, r] /. substprNB[rr, pp])[x];
        φF3FL[p0_, p2_, p3_][x_] := (φFL[p, r] /. substprF3[p0, p2, p3])[x];
        φGPFL[μ_, λ_][x_] := (φFL[p, r] /. substprGP[μ, λ])[x];
```

The functions φ where (p, r) is evaluated numerically can be plotted only after applying Evaluate to φ:

```
In[•]:= Plot[Evaluate[φBinFL[10, 0.11][x]], {x, 0, 1}]
```

Out[•]=

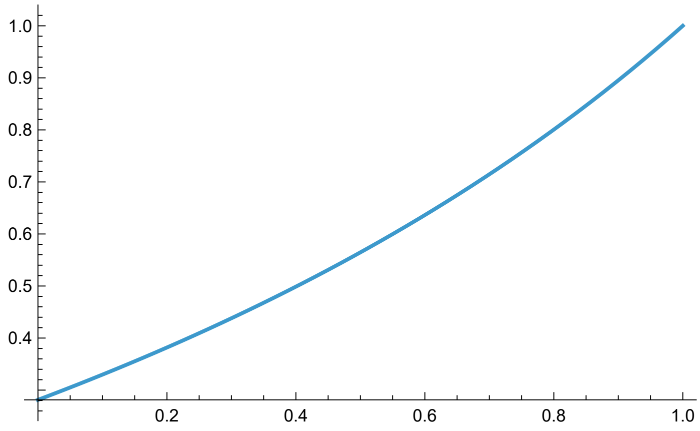

2. Poisson distribution

We recall that the basic definitions are given in Section 1

2.1. $f_{\mathrm{Poi}}(x)$ and Figure 4.1

We define

```
In[•]:= fPoi[m_][x_] := Log[φPoi[m][x]] - Log[φPoiFL[m][x]]
```

In[ ]:=
```
Plot[fPoi[1.5][x], {x, 0, 1}, PlotRange → All]
```

Out[ ]=

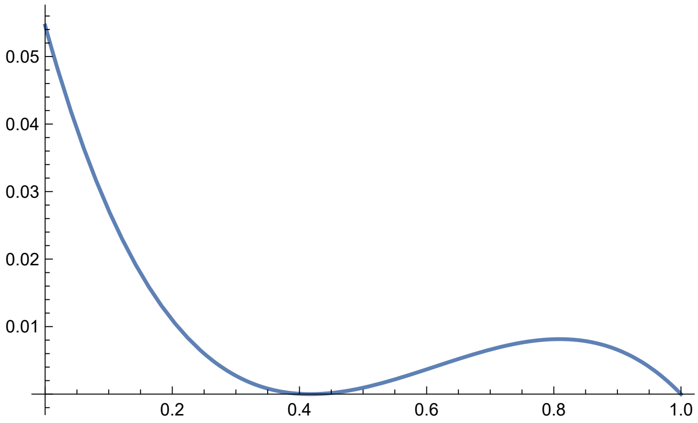

2.2. Some elements of the proof of Theorem 4.1

In[ ]:=
```
Simplify[D[fPoi[m][x], {x, 1}] /. x → 1]
```

Out[ ]=

$$m + \frac{1}{\text{ProductLog}[-e^{-m}\, m]}$$

In[ ]:=
```
Simplify[m + 1/ProductLog[-e^-m m] - (m - gammaPoi[m]^(-1))]
```

$$\text{Simplify}\left[m + \frac{1}{\text{ProductLog}[-e^{-m}\, m]} - (m - \text{gammaPoi}[m]\,{}^\wedge(-1))\right]$$

Out[ ]=

0

In[ ]:=
```
Factor[D[fPoi[m][x], {x, 2}] /. x → PinfPoi[m]]
```

Out[ ]=

$$-\frac{m^2\,(2 - m + \text{ProductLog}[-e^{-m}\, m])}{m + \text{ProductLog}[-e^{-m}\, m]}$$

The following is not a proof, but illustrates what should be proved :

In[ ]:=

$$\text{Plot}\left[-\frac{m^2\,(2 - m + \text{ProductLog}[-e^{-m}\, m])}{m + \text{ProductLog}[-e^{-m}\, m]},\ \{m, 1.0000001, 2\}\right]$$

Out[ ]=

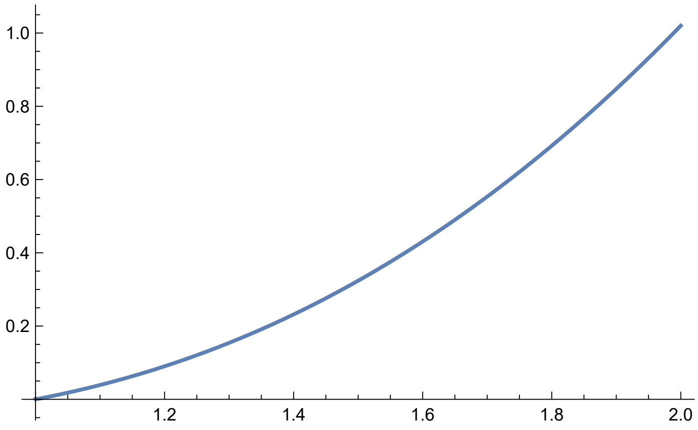

The following is the left hand side of (4.10) :

In[•]:=
```
Factor[p + 2 r /. substprPoi[m]]
```
Out[•]=

$$\frac{(m - 2\,\text{ProductLog}[-e^{-m}\,m])\,(1 + \text{ProductLog}[-e^{-m}\,m])}{m - \text{ProductLog}[-e^{-m}\,m]^2}$$

This is from where it comes :

In[•]:=
```
Simplify[D[-Log[φFL[p, r][x]], {x, 2}] /. x → PinfFL[p, r]]
```
Out[•]=

$$\frac{(-1 + p)\,p^2\,(-1 + p + 2\,r)}{(-1 + r)^2\,r^2}$$

Finally, we confirm (4.12):

In[•]:=
```
d[x_] := 3/4 (2 p x (1 - p - r) - (1 - p - r - r p))^2 + 1/4 (1 - p)^2 (1 - r)^2
```

In[•]:=
```
Simplify[D[Log[φFL[p, r][x]], {x, 3}] - 2 (1 - p) (1 - r) d[x] / ((r (1 - x) + (1 - p) x)^3 (1 - p x)^3)]
```
Out[•]=
```
0
```

2.3. Comparison with Pollak’s and Agresti's methods applied to the Poisson distribution

2.3.1. Pollak' s (1971) method and application to the Poisson distribution

In our notation, Pollak ' s eq (5.2) yields (3.18) in the ms :

$$\frac{\text{Pinf} - \text{Pn}}{\gamma\text{^}n} \leq \frac{2\,(1 - \gamma)\,\text{Pinf}}{2\,(1 - \gamma) + (1 - \gamma\text{^}n)\,\text{Pinf}\,\varphi''[\text{Pinf}]\,/\,\gamma} =: (\bar{d})^{(n)}$$

This leads to the following lower bound for Pn (where gam= $\gamma = \varphi'$[Pinf] and scndder = φ''[Pinf]):

In[•]:=
```
boundPnPollak[Pinf_, gam_, scndder_, n_] :=
  Pinf (1 - gam^n) (2 gam (1 - gam) + Pinf scndder) / (2 gam (1 - gam) + (1 - gam^n) Pinf scndder)
```

For the Poisson distribution we have

In[•]:=
```
FullSimplify[
  {PinfPoi[m], gammaPoi[m], D[φPoi[m][x], {x, 2}] /. x → PinfPoi[m]}, Assumptions → m > 1]
```
Out[•]=

$$\left\{-\frac{\text{ProductLog}[-e^{-m}\,m]}{m},\ -\text{ProductLog}[-e^{-m}\,m],\ -m\,\text{ProductLog}[-e^{-m}\,m]\right\}$$

Therefore, we obtain (4.13) in the ms :

In[•]:=
```
FullSimplify[2 (1 - γ) Pinf / (2 (1 - γ) + (1 - γ^n) Pinf scndder / γ) /. scndder → m γ /. m → γ / Pinf]
```
Out[•]=

$$\frac{2\,\text{Pinf}\,(-1 + \gamma)}{-2 + \gamma + \gamma^{1+n}}$$

This equals (4.13) :

In[◦]:= Simplify$\left[\frac{2\,\text{Pinf}\,(-1+\gamma)}{-2+\gamma+\gamma^{1+n}} - \frac{\text{Pinf}}{1+\frac{\gamma\,(1-\gamma\hat{\ }n)}{2\,(1-\gamma)}}\right]$

Out[◦]=

0

Pollak' s lower bound for Pn becomes $\frac{\text{PinfPoi}\,(1-\gamma\text{Poi}^{n})}{1-\frac{\gamma\text{Poi}^{n+1}}{2-\gamma\text{Poi}}}$:

In[◦]:= FullSimplify$\left[\text{boundPnPollak}[\text{PinfPoi}, \gamma\text{Poi}, \gamma\text{Poi}\hat{\ }2/\text{PinfPoi}, n] - \frac{\text{PinfPoi}\,(1-\gamma\text{Poi}^{n})}{1-\frac{\gamma\text{Poi}^{n+1}}{2-\gamma\text{Poi}}}\right]$

Out[◦]=

0

2.3.2. Agresti' s (1974) method for deriving bounds for the supercritical case from bounds for the subcritical case, and application to the Poisson distribution

Agresti suggests to derive bounds for the supercritical case from bounds for the subcritical case by the relation
*φ*sub[x]=*φ*[x Pinf]/Pinf, where *φ*sub is the subcritical pgf:

In[◦]:= $\varphi\text{Poisub}[m\_][x\_] := \frac{\varphi\text{Poi}[m][x\,\text{PinfPoi}[m]]}{\text{PinfPoi}[m]}$

where m > 1 and *φ*Poisub[m][x] is a subcritical generating function (our notation differs from his)

The starting point is his Theorem 2 (bounds for the Poisson distribution in the (sub)critical case; λ = mean ≤ 1); the same bound was derived by Seneta 1967, p.474).

The relation between his λ and our m is the following . His λ is:

In[◦]:= FullSimplify[D[φPoisub[m][x], x] /. x → 1]

Out[◦]=

$-\text{ProductLog}[-e^{-m}\,m]$

This is precisely our gamma:

In[◦]:= gammaPoi[m]

Out[◦]=

$-\text{ProductLog}[-e^{-m}\,m]$

Therefore, we define:

In[◦]:= $\lambda\text{Ag}[m\_] := -\text{ProductLog}[-e^{-m}\,m]$

Agresti' s lower bound for Pn (already derived by Seneta 1967, p. 474):

We follow Agresti’s definitions in his Theorem 2 for the subcritical case, where (b1,c1) and (b2,c2) are the parameters that he uses for the parameterization of *φ*FL for the lower and upper bound, respectively (see his eq 2.1):

```
In[•]:= b1AgrestPoi[λ_] := λ (2/(λ + 2))^2;
        c1AgrestPoi[λ_] := λ/(λ + 2);
```

Agresti's upper bound for Pn:

```
In[•]:= b2AgrestPoi[λ_] := (1 - Exp[-λ])^2/λ;
        c2AgrestPoi[λ_] := (λ + Exp[-λ] - 1)/λ;
```

Now we compute our parameter (π, ρ) for φFL, already in terms of m :

```
In[•]:= pFL1Poi[m_] := c1AgrestPoi[λAg[m]];
        rFL1Poi[m_] := (1 - b1AgrestPoi[λAg[m]] - c1AgrestPoi[λAg[m]])/(1 - c1AgrestPoi[λAg[m]]);
        pFL2Poi[m_] := c2AgrestPoi[λAg[m]];
        rFL2Poi[m_] := (1 - b2AgrestPoi[λAg[m]] - c2AgrestPoi[λAg[m]])/(1 - c2AgrestPoi[λAg[m]]);
```

The Agresti's lower and upper bounds for Pn in the subcritical case are

```
In[•]:= φFLAg1Poi[m_][x_] := φFL[pFL1Poi[m], rFL1Poi[m]][x];
        φFLAg2Poi[m_][x_] := φFL[pFL2Poi[m], rFL2Poi[m]][x];
```

The mean in the subcritical case is gammaPoi:

```
In[•]:= Plot[{φFLAg1Poi[1.2][x], φPoi[gammaPoi[1.2]][x], φFLAg2Poi[1.2][x]},
         {x, 0, 1}, PlotStyle → {Blue, Black, Red}]
```

Out[•]=

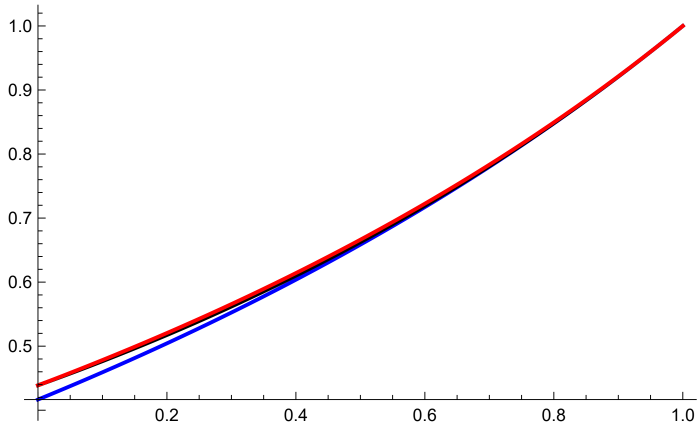

From this, we obtain by the duality relation above, the lower and upper bounds for φPoi[m][x] :

```
In[•]:= φFLAg1PoiSup[m_][z_] := PinfPoi[m] × φFLAg1Poi[m][z / PinfPoi[m]];
        φFLAg2PoiSup[m_][z_] := PinfPoi[m] × φFLAg2Poi[m][z / PinfPoi[m]];
```

In the ms, $P^{\infty} \varphi_{sub,L}(x / P^{\infty}) = \varphi$FLAg1PoiSup (x)

φFLAg1PoiSup[m][z] and φPoi[m][z] have the same 1st derivative at 1 and the same 1st and 2nd

derivatives at z = PinfPoi (for φFLAg2PoiSup[m][z] and φPoi[m][z] this is not the case for the 2nd derivative):

```
In[∘]:= Simplify[D[φFLAg1PoiSup[m][z], {z, 1}] /. z → PinfPoi[m]]
Out[∘]=
-ProductLog[-e^-m m]

In[∘]:= FullSimplify[D[φPoi[m][z], {z, 1}] /. z → PinfPoi[m]]
Out[∘]=
-ProductLog[-e^-m m]

In[∘]:= FullSimplify[D[φFLAg1PoiSup[m][z], {z, 2}] /. z → PinfPoi[m], Assumptions → m > 1]
Out[∘]=
-m ProductLog[-e^-m m]

In[∘]:= FullSimplify[D[φPoi[m][z], {z, 2}] /. z → PinfPoi[m], Assumptions → m > 1]
Out[∘]=
-m ProductLog[-e^-m m]

In[∘]:= {PinfPoi[m], gammaPoi[m]}
Out[∘]=
```

$$\left\{-\frac{\text{ProductLog}[-e^{-m}\,m]}{m},\ -\text{ProductLog}[-e^{-m}\,m]\right\}$$

```
In[∘]:= FullSimplify[D[φFLAg1Poi[m][z], {z, 1}] /. z → 1]
Out[∘]=
-ProductLog[-e^-m m]

In[∘]:= FullSimplify[D[φFLAg2PoiSup[m][z], {z, 1}] /. z → PinfPoi[m]]
Out[∘]=
-ProductLog[-e^-m m]

In[∘]:= FullSimplify[D[φFLAg2PoiSup[m][z], {z, 2}] /. z → PinfPoi[m], Assumptions → m > 1]
Out[∘]=
```

$$2\,m\left(-1+\frac{\text{ProductLog}[-e^{-m}\,m]}{-1+e^{\text{ProductLog}[-e^{-m}\,m]}}\right)$$

The upper bound is red, the lower bound is blue:

```
In[∘]:= Plot[{φFLAg1PoiSup[1.2][x], φPoi[1.2][x], φFLAg2PoiSup[1.2][x]},
  {x, 0, 1}, PlotStyle → {Blue, Black, Red}]
Out[∘]=
```

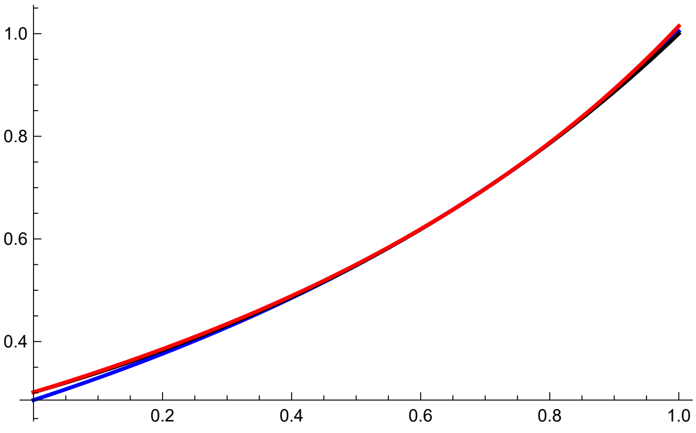

In[•]:= φFLAg1PoiSup and φFLAg2PoiSup are not generating functions because

```
In[•]:= {φFLAg1PoiSup[1.2][1], φFLAg2PoiSup[1.2][1]}
Out[•]=
{1.00455, 1.01489}
```

Plot differences :

```
In[•]:= Plot[{φPoi[1.2][x] - φFLAg1PoiSup[1.2][x], φFLAg2PoiSup[1.2][x] - φPoi[1.2][x]},
  {x, 0, 1}, PlotStyle → {Blue, Red}]
Out[•]=
```

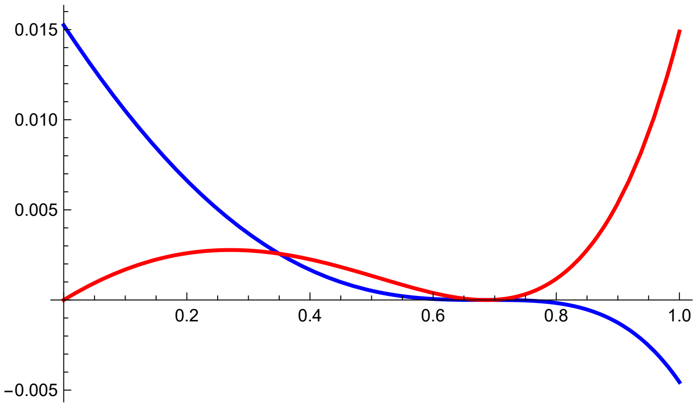

Obviously, φFLAg1PoiSup is a lower bound only if x ≤ Pinf

Comparison of relative errors of Agresti’s lower bound (blue) with our lower bound (red) for φPoi:

```
In[•]:= Plot[{(φPoi[1.2][x] - φFLAg1PoiSup[1.2][x]) / φPoi[1.2][x],
   (φPoi[1.2][x] - φPoiFL[1.2][x]) / φPoi[1.2][x]}, {x, 0, 1}, PlotStyle → {Blue, Red}]
Out[•]=
```

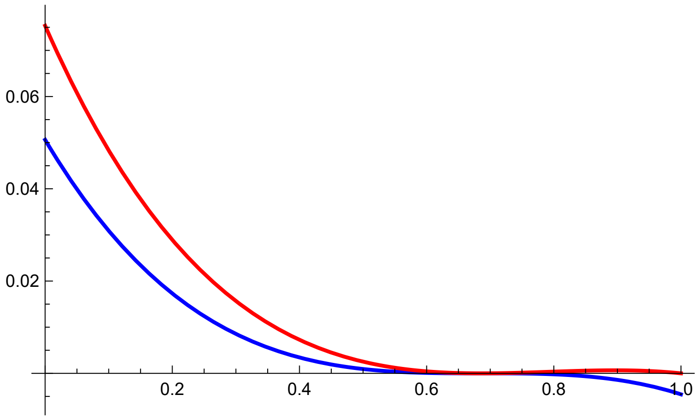

In[∘]:=
```
Plot[{(φPoi[1.05][x] - φFLAg1PoiSup[1.05][x]) / φPoi[1.05][x],
  (φPoi[1.05][x] - φPoiFL[1.05][x]) / φPoi[1.05][x]}, {x, 0, 1}, PlotStyle → {Blue, Red}]
```
Out[∘]=

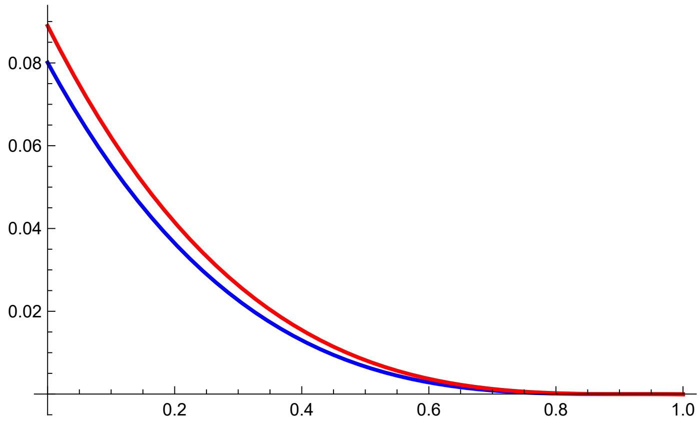

Numerical evaluation of the extinction probabilities up to generation n
(exact = φPoi, our bound = φPoiFL, Agresti' s lower bound = φFLAg1PoiSup):

In[∘]:=
```
NestList[φPoi[1.2], 0, 20]
```
Out[∘]=
```
{0, 0.301194, 0.43233, 0.506007, 0.552782, 0.584697, 0.607524,
 0.624396, 0.637166, 0.647005, 0.65469, 0.660755, 0.665582, 0.669448,
 0.672561, 0.675078, 0.67712, 0.678782, 0.680136, 0.681243, 0.682148}
```

In[∘]:=
```
NestList[φPoiFL[1.2], 0, 20]
```
Out[∘]=
```
{0, 0.278503, 0.413114, 0.49128, 0.541565, 0.576075, 0.600823,
 0.619134, 0.632998, 0.643681, 0.652023, 0.658605, 0.663841, 0.668035,
 0.671411, 0.67414, 0.676354, 0.678155, 0.679623, 0.680822, 0.681802}
```

In[∘]:=
```
NestList[φFLAg1PoiSup[1.2], 0, 20]
```
Out[∘]=
```
{0, 0.285948, 0.420447, 0.497474, 0.546616, 0.580158, 0.60412,
 0.6218, 0.63516, 0.645437, 0.653453, 0.659771, 0.664795, 0.668815,
 0.67205, 0.674664, 0.676784, 0.678508, 0.679913, 0.68106, 0.681998}
```

In[∘]:=
```
NestList[φPoi[1.02], 0, 20]
```
Out[∘]=
```
{0, 0.360595, 0.520902, 0.613436, 0.674155, 0.717228, 0.749441,
 0.774475, 0.794505, 0.810905, 0.824583, 0.836168, 0.846108, 0.85473,
 0.86228, 0.868946, 0.874874, 0.88018, 0.884957, 0.889279, 0.893209}
```

In[∘]:=
```
NestList[φPoiFL[1.02], 0, 20]
```
Out[∘]=
```
{0, 0.327453, 0.490877, 0.588819, 0.654056, 0.700618, 0.735513,
 0.762634, 0.784314, 0.802039, 0.816797, 0.829273, 0.839957, 0.849207,
 0.857293, 0.864419, 0.870746, 0.876401, 0.881483, 0.886075, 0.890243}
```

In[∘]:=
```
NestList[φFLAg1PoiSup[1.02], 0, 20]
```
Out[∘]=
```
{0, 0.328833, 0.492411, 0.590274, 0.655389, 0.701828, 0.736614,
 0.763638, 0.785234, 0.802884, 0.817578, 0.829998, 0.840631, 0.849837,
 0.857883, 0.864973, 0.871268, 0.876892, 0.881948, 0.886515, 0.890661}
```

2.3.3. Comparison of the relative errors of Sn resulting from the different bounds

Our lower bound (3.7) for PnPoi is $\frac{\text{PinfPoi } (1-\gamma\text{Poi}^n)}{1-\gamma\text{Poi}^n \text{ PinfPoi}}$

In[ ]:=
```
boundPnRB[Pinf_, gam_, n_] := Pinf (1 - gam^n) / (1 - gam^n Pinf)
```

The simple bound resulting from (3.15) in the ms is :

In[ ]:=
```
boundPnSimple[Pinf_, gam_, n_] := Pinf (1 - gam^n)
```

Pollak' s and Agresti' s lower bounds are indeed identical (as already noted by Agresti for the subcritical case):

In[ ]:=
```
FullSimplify[Table[Nest[φFLAg1PoiSup[m], 0, k] -
   boundPnPollak[PinfPoi[m], gammaPoi[m], m gammaPoi[m], k], {k, 1, 10}]]
```

Out[ ]=
```
{0, 0, 0, 0, 0, 0, 0, 0, 0, 0}
```

In principle, the iterates of φFLAg1PoiSup[m] can be computed explicitly using eq (2.11) in the main text . However, we abstain from it because Agresti already noted equality with Pollak’s bound.

We define the relative errors of the survival probabilities Sn for the three different methods (recall that φitPoi[m, n][0] = Pn, the n - fold iteration of φPoi) :

In[ ]:=
```
relErrSnSimple[m_, nmax_] :=
  (φitPoi[m, nmax][0] - Table[boundPnSimple[PinfPoi[m], gammaPoi[m], k],
     {k, 1, nmax}]) / (1 - φitPoi[m, nmax][0]);
relErrSnRB[m_, nmax_] :=
  (φitPoi[m, nmax][0] - Table[boundPnRB[PinfPoi[m], gammaPoi[m], k], {k, 1, nmax}]) /
   (1 - φitPoi[m, nmax][0]);
relErrSnPollak[m_, nmax_] :=
  (φitPoi[m, nmax][0] - Table[boundPnPollak[PinfPoi[m], gammaPoi[m],
      m gammaPoi[m], k], {k, 1, nmax}]) / (1 - φitPoi[m, nmax][0]);
```

Data for Table 4.1 :

m = 1.5:

In[ ]:=
```
{relErrSnSimple[1.5, 200][[{1, 5, 10, 20, 50, 100, 200}]],
 relErrSnRB[1.5, 200][[{1, 5, 10, 20, 50, 100, 200}]],
 relErrSnPollak[1.5, 200][[{1, 5, 10, 20, 50, 100, 200}]]}
```

Out[ ]=

$\{\{0.0862577, 0.0294636, 0.00306645, 0.0000284699, 2.22394\times10^{-11}, 0., 0.\},$
$\{0.0152574, 0.00346553, 0.000340607, 3.14371\times10^{-6}, 2.45566\times10^{-12}, 0., 0.\},$
$\{0.00616883, 0.000977183, 0.0000900595, 8.25513\times10^{-7}, 6.44823\times10^{-13}, 0., 0.\}\}$

m = 1.1:

In[ ]:=
```
{relErrSnSimple[1.1, 200][[{1, 5, 10, 20, 50, 100, 200}]],
 relErrSnRB[1.1, 200][[{1, 5, 10, 20, 50, 100, 200}]],
 relErrSnPollak[1.1, 200][[{1, 5, 10, 20, 50, 100, 200}]]}
```

Out[ ]=

$\{\{0.383188, 0.986854, 0.94154, 0.473267, 0.0282291, 0.000207065, 1.09912\times10^{-8}\},$
$\{0.0420279, 0.037154, 0.020838, 0.00704969, 0.00034832, 2.5303\times10^{-6}, 1.34303\times10^{-10}\},$
$\{0.0341988, 0.026153, 0.0132144, 0.00403586, 0.000187971, 1.36103\times10^{-6}, 7.224\times10^{-11}\}\}$

m = 1.02:

In[ ]:=
```
{relErrSnSimple[1.02, 200][[{1, 5, 10, 20, 50, 100, 200}]],
 relErrSnRB[1.02, 200][[{1, 5, 10, 20, 50, 100, 200}]],
 relErrSnPollak[1.02, 200][[{1, 5, 10, 20, 50, 100, 200}]]}
```

Out[ ]=

```
{{0.534289, 2.214, 3.71055, 5.40513, 5.58914, 2.8017, 0.432376},
 {0.051832, 0.0587394, 0.0443885, 0.0277688, 0.0104987, 0.00316334, 0.000395488},
 {0.0496735, 0.0544576, 0.039935, 0.0238573, 0.00826737, 0.0023323, 0.000281982}}
```

3. Binomial distribution

We recall that the basic definitions are given in Section 1

3.1. We switch to the parameters n and z = $\text{PinfBin}^{1/n}$

The z here corresponds to ξ in the ms.

The we obtain:

In[ ]:= Simplify[Solve[(1 - p + p z^n) == z, p]]

Out[ ]=

$$\left\{\left\{p \to \frac{-1+z}{-1+z^n}\right\}\right\}$$

In[ ]:=

$$\text{pzn} = \frac{1-z}{1-z^n};$$

In[ ]:= Simplify[D[φBin[n, pzn][x], {x, 1}] /. x → z^n]

Out[ ]=

$$\frac{n\,(-1+z)\,z^{-1+n}}{-1+z^n}$$

Therefore, we have

In[ ]:=

$$\text{gammaBinz} = \frac{n\,(1-z)\,z^n}{z\,(1-z^n)};$$

We use (3.3) and (3.4) in the ms to calculate the parameters π and ρ for the fractional linear bound :

In[ ]:= Simplify[Solve[φFL[p, r][z^n] == z^n && (D[φFL[p, r][x], x] /. x → z^n) == gammaBinz, {p, r}]]

Out[ ]=

$$\left\{\{p \to z^{-n}, r \to 1\}, \left\{p \to \frac{z - n z^n + (-1+n)\,z^{1+n}}{z - n z^{2n} - z^{1+n} + n z^{1+2n}}, r \to \frac{z^n\left(z - n z^n + (-1+n)\,z^{1+n}\right)}{z - n z^{2n} - z^{1+n} + n z^{1+2n}}\right\}\right\}$$

In[ ]:=
```
substprBinz = {p → (z (1 - z^n + n z^n) - n z^n)/(z (1 - z^n + n z^(2n)) - n z^(2n)), r → z^n (z (1 - z^n + n z^n) - n z^n)/(z (1 - z^n + n z^(2n)) - n z^(2n))};
```

3.2. Proof of Theorem 4.4 (Appendix A in the ms)

We define (see eq (A.1) in the ms):

In[ ]:=
```
xit = Sum[z^k, {k, 0, n - 1}];
v[n_, z_][x_] := ((x - z^n) (1 - z))/(z (1 - z^n))
```

In[ ]:=
```
Simplify[v[n, z][x] - (x - z^n)/(z xit)]
```

Out[ ]=

$$0$$

In[ ]:=
```
Simplify[{v[n, z][0], v[n, z][z^n], v[n, z][1]}]
```

Out[ ]=

$$\left\{-\frac{(-1+z)\, z^{-1+n}}{-1+z^n},\ 0,\ -1+\frac{1}{z}\right\}$$

In[ ]:=
```
Simplify[Solve[v[n, z][x] == v, x]]
```

Out[ ]=

$$\left\{\left\{x \to z^n + \frac{v\, z\, (-1+z^n)}{-1+z}\right\}\right\}$$

The following confirms (A.4) :

In[ ]:=
```
FullSimplify[φBin[n, (1 - z)/(1 - z^n)][z^n + (v z (-1 + z^n))/(-1 + z)]]
```

Out[ ]=

$$((1+v)\, z)^n$$

The following confirms (A.5):

In[ ]:=
```
FullSimplify[(φFL[p, r][z^n + (v z (-1 + z^n))/(-1 + z)] /. substprBinz) -
  z^n ((1 - z) (1 - z^n) + v (n (1 - z) - z (1 - z^n)))/((1 - z) (1 - z^n) + v (n z^n (1 - z) - z (1 - z^n)))]
```

Out[ ]=

$$0$$

The following is fBin(v) in eq (A.6) :

In[ ]:=
```
fBin[n_, z_][v_] := (1 + v)^n ((1 - z) (1 - z^n) + v (n z^n (1 - z) - z (1 - z^n))) -
   ((1 - z) (1 - z^n) + v (n (1 - z) - z (1 - z^n)))
```

The following is fBin in the first line of eq (A.7) :

In[ ]:=
```
fBinA[n_, z_][v_] := ((((1 + v)^n - 1) (1 - (1 + v) z) (1 - z^n)) - n v (1 - z) (1 - (1 + v)^n z^n))
```

```
In[•]:= Simplify[fBin[n, z][v] - fBinA[n, z][v]]
Out[•]=
0
```

Defining

```
In[•]:= fhatBin[n_, z_][v_] :=
         ((1 + v)^n - 1) Sum[z^k, {k, 0, n - 1}]/(1 - z) - (n v)/(1 - z) Sum[(1 + v)^k z^k, {k, 0, n - 1}]
```

as in (A.8), we obtain (A.7):

```
In[•]:= FullSimplify[fBinA[n, z][v] - (1 - z)^2 (1 - (1 + v) z) fhatBin[n, z][v]]
Out[•]=
0
```

Define the following coefficients of v^j occurring in (A.9b) and (A.9c):

```
In[•]:= coeff1serz[n_, j_] := Binomial[n, j + 1] (Sum[(k + 1) z^k, {k, 0, n - 1}] + n z^n / (1 - z));
        coeff2serz[n_, j_] :=
          n (Sum[Binomial[j + k + 1, k] z^(j + k), {k, 0, n - j - 1}] + Binomial[n, j + 1] z^n 1/(1 - z));
```

Then fhatBin in (A.9b) and (A.9c) is

```
In[•]:= fhatBinA[n_, z_][v_] :=
         v Sum[v^j coeff1serz[n, j], {j, 0, n - 1}] - v Sum[v^j coeff2serz[n, j], {j, 0, n - 1}]
```

The following confirms this:

```
In[•]:= Simplify[fhatBin[5, z ][v] - fhatBinA[5, z ][v]]
Out[•]=
0
```

```
In[•]:= Simplify[fhatBin[16, z ][v] - fhatBinA[16, z ][v]]
Out[•]=
0
```

Finally, we define the coefficient of v^j z^k occurring in (A.10) :

```
In[•]:= coeffvza[n_, j_, k_] := Binomial[n, j] (k + 1) - n Binomial[k + 1, j]
```

```
In[•]:= fhatBinB[n_, z_][v_] :=
          v Sum[v^j Sum[z^k coeffvza[n, j + 1, k], {k, 0, n - 1}], {j, 0, n - 1}];
```

```
In[•]:= Simplify[fhatBinB[15, z][v] - fhatBinfinal[15, z][v]]
Out[•]=
0
```

The following is (A.11)

```
In[•]:= coeffvz[n_, j_, k_] := Binomial[n, j + 2] (k + 1) - n Binomial[k + 1, j + 2];
        fhatBinfinal[n_, z_][v_] :=
          v^2 Sum[v^j Sum[z^k coeffvz[n, j, k], {k, 0, n - 2}], {j, 0, n - 2}];
```

Check:

In[∘]:=
```
Simplify[fhatBin[15, z][v] - fhatBinfinal[15, z][v]]
```
Out[∘]=
```
0
```
In[∘]:=
```
Factor[fhatBin[10, z][v] - fhatBinfinal[10, z][v]]
```
Out[∘]=
```
0
```
Graphs :

In[∘]:=
```
0.9^5
```
Out[∘]=
```
0.59049
```
In[∘]:=
```
Plot[fBin[5, 0.9][v[5, 0.9][x]], {x, 0, 1}, PlotRange → {-10^(-5), 10^(-3)}]
```
Out[∘]=

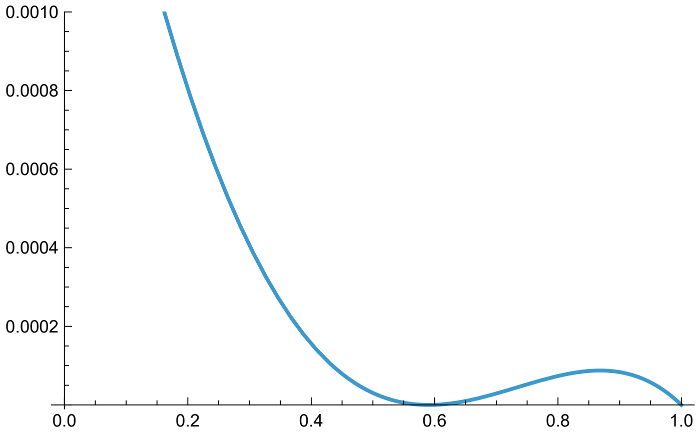

In[∘]:=
```
Plot[fhatBinfinal[5, 0.9][v[5, 0.9][x]], {x, 0, 1}, PlotRange → All]
```
Out[∘]=

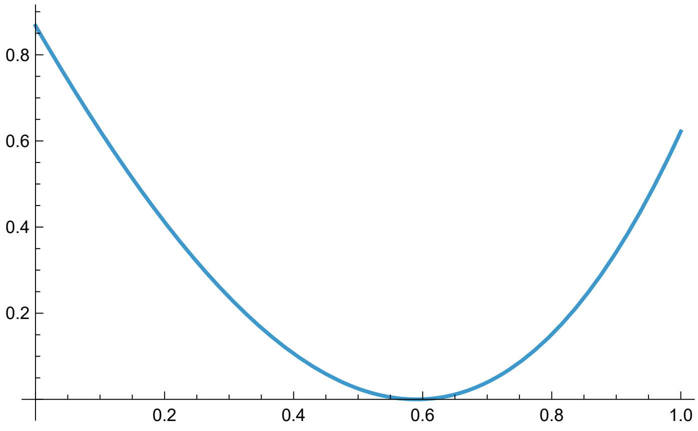

Ultimate numerical check :

In[•]:=
```
Plot[{fBin[5, 0.9][v[5, 0.9][x]],
   (1 - 0.9)^2 (1 - (1 + v[5, 0.9][x]) 0.9) fhatBinfinal[5, 0.9][v[5, 0.9][x]]},
  {x, 0, 1}, PlotRange → All, PlotStyle → {Blue, Dashed}]
```

Out[•]=

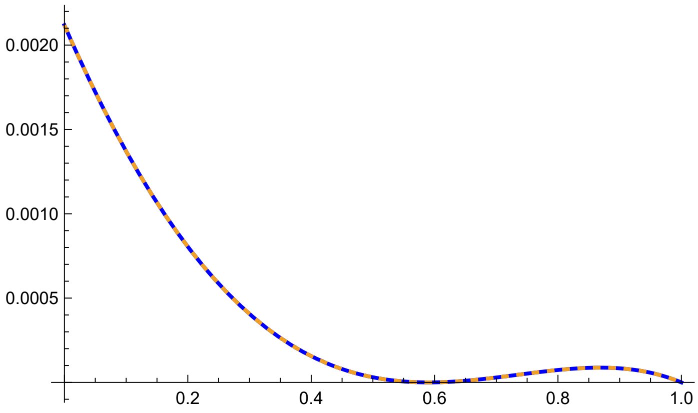

The following yields (A .12) :

In[•]:=
```
coeffvzratio[n_, j_, k_] := (Binomial[n, j + 2] (k + 1)) / (n Binomial[k + 1, j + 2])
coeffvzsimp[n_, j_, k_] := Product[n - 1 - i, {i, 0, j}] / Product[k - i, {i, 0, j}]
```

In[•]:=
```
Simplify[
  Table[Table[coeffvzratio[10, j, k] - coeffvzsimp[10, j, k], {j, 0, k - 1}], {k, 1, 8}]]
```

Out[•]=
```
{{0}, {0, 0}, {0, 0, 0}, {0, 0, 0, 0}, {0, 0, 0, 0, 0},
 {0, 0, 0, 0, 0, 0}, {0, 0, 0, 0, 0, 0, 0}, {0, 0, 0, 0, 0, 0, 0, 0}}
```

Assume v < 0

We use v > -1/n. The following confirm (A.14) and (A.15):

In[•]:=
```
FullSimplify[(coeffvz[n, j, k] - 1/n coeffvz[n, j + 1, k]) -
  (Binomial[n, j + 2] (k + 1) (1 - 1/n (n - j - 2)/(j + 3)) - n Binomial[k + 1, j + 2] (1 - 1/n (k - j - 1)/(j + 3)))]
```

Out[•]=
```
0
```

In[•]:=
```
fac2[n_, j_, k_] := (n (j + 3) - n + j + 2) / (n (j + 3) - k + j + 1)
```

In[•]:=
```
FullSimplify[
  (Binomial[n, j + 2] (k + 1) (1 - 1/n (n-j-2)/(j+3))) / (n Binomial[k + 1, j + 2] (1 - 1/n (k-j-1)/(j+3))) / (coeffvzsimp[n, j, k] × fac2[n, j, k])]
```

Out[•]=
```
1
```

In[ ]:=
```
Series[1/(v^2) fhatBinfinal[7, z][v], {v, 0, 7}]
```

Out[ ]=

$$\left(21+35 z+42 z^2+42 z^3+35 z^4+21 z^5\right)+\left(35+70 z+98 z^2+112 z^3+105 z^4+70 z^5\right) v+\left(35+70 z+105 z^2+133 z^3+140 z^4+105 z^5\right) v^2+\left(21+42 z+63 z^2+84 z^3+98 z^4+84 z^5\right) v^3+\left(7+14 z+21 z^2+28 z^3+35 z^4+35 z^5\right) v^4+\left(1+2 z+3 z^2+4 z^3+5 z^4+6 z^5\right) v^5+O[v]^8$$

The following confirms (A.16) :

In[ ]:=
```
Simplify[Reduce[(n - 1)/k fac2[n, j, k] > 1 && 1 ≤ k ≤ n - 2 && 0 ≤ j ≤ k - 1 && n ≥ 2],
 Assumptions → 1 ≤ k ≤ n - 2 && 0 ≤ j ≤ k - 1 && n ≥ 2]
```

Out[ ]=

True

4. Negative binomial distribution

We recall that the basic definitions are given in Section 1

4.1. We switch to the parameters r and ζ = PinfNB$^{1/r}$

The we obtain:

```
Simplify[Solve[φNB[r, p][ζ^r] == ζ^r, p]]
```

Out[ ]=

$$\left\{\left\{p \to \frac{\zeta\,(-1+\zeta^n)}{-1+\zeta^{1+n}}\right\}\right\}$$

In[ ]:=
```
pζr = ζ (ζ^r - 1)/(ζ^(1+r) - 1);
```

In[ ]:=
```
FullSimplify[D[φNB[r, pζr][x], {x, 1}] /. x → ζ^r, Assumptions → ζ > 0 && r > 1]
```

Out[ ]=

$$\frac{r\,(-1+\zeta)\,\zeta^r}{-1+\zeta^r}$$

Therefore, we have

In[ ]:=
```
gammaNBζ = r (1 - ζ) ζ^r/(1 - ζ^r);
```

We use (4.3) and (4.4) in the ms to calculate the parameters π and ρ for the fractional linear bound :

In[ ]:=
```
FullSimplify[
 Solve[φFL[πNB, ρNB][ζ^r] == ζ^r && (D[φFL[πNB, ρNB][x], x] /. x → ζ^r) == gammaNBζ,
  {πNB, ρNB}], Assumptions → ζ > 0 && r > 1]
```

Out[ ]=

$$\left\{\left\{\pi NB \to \zeta^{-r},\ \rho NB \to 1\right\},\ \left\{\pi NB \to \frac{1+(-1+r\,(-1+\zeta))\,\zeta^r}{1+\zeta^r\,(-1+r\,(-1+\zeta)\,\zeta^r)},\ \rho NB \to \frac{\zeta^r+(-1+r\,(-1+\zeta))\,\zeta^{2r}}{1+\zeta^r\,(-1+r\,(-1+\zeta)\,\zeta^r)}\right\}\right\}$$

In[•]:=
$$\text{substprBN}\xi = \left\{\pi\text{NB} \to \frac{1 - (1 + r\,(1 - \xi))\,\xi^{r}}{1 - \xi^{r}\,(1 + r\,(1 - \xi)\,\xi^{r})},\ \rho\text{NB} \to \xi^{r}\,\frac{1 - (1 + r\,(1 - \xi))\,\xi^{r}}{1 - \xi^{r}\,(1 + r\,(1 - \xi)\,\xi^{r})}\right\};$$

4.2. Proof of Theorem 4.6 (Appendix B in the ms)

As in (B .1) we define

In[•]:=
```
ξit[r_, ξ_] := Sum[ξ^k, {k, 0, r - 1}];
```
$$\text{yr}[r\_, \xi\_][x\_] := \frac{\xi\verb|^|r - x}{\xi\text{it}[r, \xi]}$$

We note that

In[•]:=
```
Simplify[{yr[r, ξ][0], yr[r, ξ][ξ^r], yr[r, ξ][1]}]
```

Out[•]=
$$\left\{\frac{(-1 + \xi)\,\xi^{r}}{-1 + \xi^{r}},\ 0,\ -1 + \xi\right\}$$

and we obtain (B.3) and (B.4):

In[•]:=
$$\text{FullSimplify}\left[(\varphi\text{FL}[\pi\text{NB}, \rho\text{NB}][x]\ /.\ \text{substprBN}\xi) - \left(\xi\verb|^|r\,\frac{1 - x - r\,\text{yr}[r, \xi][x]}{1 - x - r\,\xi\verb|^|r\,\text{yr}[r, \xi][x]}\right),\ \text{Assumptions} \to 0 < \xi < 1\ \&\&\ r > 1\ \&\&\ 0 < x < 1\right]$$

Out[•]=
```
0
```

In[•]:=
$$\text{FullSimplify}\left[\frac{p\xi r}{(1 - (1 - p\xi r)\,x)} - \frac{\xi}{(1 + \text{yr}[r, \xi][x])},\ \text{Assumptions} \to 0 < \xi < 1\ \&\&\ r > 1\ \&\&\ 0 < x < 1\right]$$

Out[•]=
```
0
```

The following is $\xi^{r}\left(\varphi\text{FL}^{-1} - \varphi\text{NB}^{-1}\right)$, see eq (B .5) :

In[•]:=
$$\text{fzetNB}[r\_, \xi\_][x\_] := \frac{1 - x - r\,\xi\verb|^|r\,\text{yr}[r, \xi][x]}{1 - x - r\,\text{yr}[r, \xi][x]} - (1 + \text{yr}[r, \xi][x])\verb|^|r;$$

Choose PinfNB = 0.7, 0.8, 0.9 and r = 6 :

In[•]:=
```
Plot[{fzetNB[6, 0.7^(1/6)][x], fzetNB[6, 0.8^(1/6)][x], fzetNB[6, 0.9^(1/6)][x]},
  {x, 0.5, 1}, PlotRange → {0, 0.001}]
```

Out[•]=

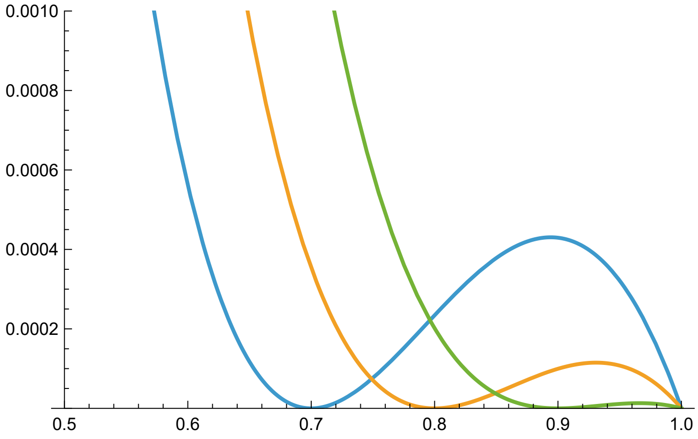

In[•]:=
```
Simplify[Solve[y == yr[r, ζ][x], x]]
```

Out[•]=

$$\left\{\left\{x \to \frac{y - y\,\zeta^r + (-1+\zeta)\,\zeta^r}{-1+\zeta}\right\}\right\}$$

In[•]:=

$$\text{substxy} = \left\{x \to \zeta^r - y\,\frac{1-\zeta^r}{1-\zeta}\right\};$$

Multiply fzetNB by (1 - x - r yr[r, ζ][x])/(1-ζ)^2 and substitute x by y (we know 1 - x - r y > 0 (see below (B .10))):

In[•]:=

$$\text{Simplify}\left[-\frac{1 - x + r\,x - r\,x\,\zeta - \zeta^r - r\,\zeta^r + x\,\zeta^r + r\,\zeta^{1+r}}{-1+\zeta^r} - \frac{1 - \zeta\text{^}r\,(1 + r\,(1-\zeta)) - x\,(1 - \zeta\text{^}r - r\,(1-\zeta))}{1 - \zeta\text{^}r}\right]$$

Out[•]=

```
0
```

In[•]:=
```
Simplify[(1 - x - r yr[r, ζ][x]) /. substxy]
```

Out[•]=

$$1 - \zeta^r + \frac{y\,(-1 + r - r\,\zeta + \zeta^r)}{-1+\zeta}$$

As in (B.7), we define

In[•]:=

$$\text{gzetNB}[r\_, \zeta\_][y\_] := \text{fzetNB}[r, \zeta]\left[\zeta^r - y\,\frac{(1-\zeta^r)}{1-\zeta}\right]\,\frac{(1-\zeta)\,(1-\zeta^r) + y\,(1 - \zeta^r - r\,(1-\zeta))}{(1-\zeta)\text{^}3}$$

Then

In[•]:=

$$\text{gzetNBA}[r\_, \zeta\_][y\_] := \frac{r\,y\,((1+y)\text{^}r - \zeta\text{^}r)\,(1-\zeta) - ((1+y)\text{^}r - 1)\,(1-\zeta\text{^}r)\,(1-\zeta+y)}{(1-\zeta)^3}$$

and

```
In[•]:= Simplify[gzetNB[r, ξ][y] - gzetNBA[r, ξ][y]]
Out[•]=
0
```

The following is (B .8) :

```
In[•]:= gzetNBAA[r_, ξ_][y_] :=
         1/(1 - ξ)^3 (y ((1 + y)^r - 1) (r (1 - ξ) - (1 - ξ^r)) - (1 - ξ) (1 - ξ^r) (((1 + y)^r - 1) - r y))

In[•]:= Simplify[gzetNB[r, ξ][y] - gzetNBAA[r, ξ][y]]
Out[•]=
0
```

Now use binomial expnasion of (1 + y)^r - 1 :

```
In[•]:= gzetNBB[r_, ξ_][y_] :=
         y^2 1/(1 - ξ)^3 (Sum[Binomial[r, j + 1] y^j, {j, 0, r - 1}] (r (1 - ξ) - (1 - ξ^r)) -
           (1 - ξ^r) (1 - ξ) Sum[Binomial[r, j + 2] y^j, {j, 0, r - 2}])

In[•]:= Simplify[gzetNB[r, ξ][y] - gzetNBB[r, ξ][y]]
Out[•]=
0
```

The following is (B.9a) :

```
In[•]:= gzetNBC[r_, ξ_][y_] := y^2 1/(1 - ξ)^2 (Sum[
           y^j (Binomial[r, j + 1] (r - (1 - ξ^r)/(1 - ξ)) - Binomial[r, j + 2] (1 - ξ^r)), {j, 0, r - 1}])

In[•]:= Simplify[gzetNB[5, ξ][y] - gzetNBB[5, ξ][y]]
Out[•]=
0
```

And here is (B.9b) :

```
In[•]:= gzetNBC[r_, ξ_][y_] := y^2 1/(1 - ξ)^2
         (Sum[y^j Binomial[r, j + 1] ((r - (1 - ξ^r)/(1 - ξ)) - (r - j - 1)/(j + 2) (1 - ξ^r)), {j, 0, r - 1}])

In[•]:= Simplify[gzetNBC[5, ξ][y] - gzetNB[5, ξ][y]]
Out[•]=
0
```

The following is cg (r, φ, ζ) used in (B.10) and defined (B.11) :

In[∘]:=
```
coeffgzetNBfinal[r_, j_, ζ_] := 1/(2 (j + 2))
  (Sum[ζ^k (k + 1) (2 r (1 + j) - (2 + j) k - 2), {k, 0, r - 2}] + ζ^(r - 1)/(1 - ζ) r (r + 1) j);
```

In[∘]:=
```
FullSimplify[1/(1 - ζ)^2 ((r - (1 - ζ^r)/(1 - ζ)) - (r - j - 1)/(j + 2) (1 - ζ^r)) - coeffgzetNBfinal[r, j, ζ]]
```

Out[∘]=
```
0
```

The following is (B.10) :

In[∘]:=
```
gzetNBfinal[r_, ζ_][y_] :=
  y^2 (Sum[y^j Binomial[r, j + 1] coeffgzetNBfinal[r, j, ζ], {j, 0, r - 1}])
```

In[∘]:=
```
FullSimplify[gzetNB[r, ζ][y] - gzetNBfinal[r, ζ][y]]
```

Out[∘]=
```
0
```

We check that coeffgzetNBfinal > 0 :

In[∘]:=
```
Simplify[Reduce[(2 r (1 + j) - (2 + j) k - 2) ≥ 2], Assumptions → 0 ≤ j ≤ r - 1 && 0 ≤ k ≤ r - 2]
```

Out[∘]=
```
True
```

This finishes the proof (if y>0).

4.3. Proof of φNBFL ≤ φNB for 0 ≤ x ≤ 1 for n = 2, 3, 4, 5, 6

We use the transformation y → u - (1 - ζ), where 1 - ζ > u > 0, i .e ., -(1 - ζ) < y < 0 or, equivalently, ζ^r < x < 1 (cf . (B .2)).
We show that $\frac{1-\zeta}{y\hat{}2}$gzetNB[r,ζ][u-(1 - ζ)] >0 .

In[∘]:=
```
Simplify[Series[(1 - ζ) gzetNB[2, ζ][y] / y^2 /. y → u - (1 - ζ), {u, 0, 2}]]
```

Out[∘]=

$u + O[u]^3$

In[∘]:=
```
Simplify[Series[(1 - ζ) gzetNB[3, ζ][y] / y^2 /. y → u - (1 - ζ), {u, 0, 3}]]
```

Out[∘]=

$(1 + 4\zeta + \zeta^2)\, u + (2 + \zeta)\, u^2 + O[u]^4$

In[∘]:=
```
Simplify[Series[(1 - ζ) gzetNB[4, ζ][y] / y^2 /. y → u - (1 - ζ), {u, 0, 4}]]
```

Out[∘]=

$(1 + 4\zeta + 10\zeta^2 + 4\zeta^3 + \zeta^4)\, u + 2\,(1 + 5\zeta + 3\zeta^2 + \zeta^3)\, u^2 + (3 + 2\zeta + \zeta^2)\, u^3 + O[u]^5$

In[∘]:=
```
Simplify[Series[(1 - ζ) gzetNB[5, ζ][y] / y^2 /. y → u - (1 - ζ), {u, 0, 5}]]
```

Out[∘]=

$(1 + 4\zeta + 10\zeta^2 + 20\zeta^3 + 10\zeta^4 + 4\zeta^5 + \zeta^6)\, u + (2 + 10\zeta + 30\zeta^2 + 20\zeta^3 + 10\zeta^4 + 3\zeta^5)\, u^2 + (3 + 18\zeta + 13\zeta^2 + 8\zeta^3 + 3\zeta^4)\, u^3 + (4 + 3\zeta + 2\zeta^2 + \zeta^3)\, u^4 + O[u]^6$

```
In[]:= Simplify[Series[(1 - ξ) gzetNB[6, ξ][y] / y^2 /. y → u - (1 - ξ), {u, 0, 6}]]
Out[]=
```

$$\left(1+4\,\xi+10\,\xi^2+20\,\xi^3+35\,\xi^4+20\,\xi^5+10\,\xi^6+4\,\xi^7+\xi^8\right)u+2\left(1+5\,\xi+15\,\xi^2+35\,\xi^3+25\,\xi^4+15\,\xi^5+7\,\xi^6+2\,\xi^7\right)u^2+3\left(1+6\,\xi+21\,\xi^2+16\,\xi^3+11\,\xi^4+6\,\xi^5+2\,\xi^6\right)u^3+2\left(2+14\,\xi+11\,\xi^2+8\,\xi^3+5\,\xi^4+2\,\xi^5\right)u^4+\left(5+4\,\xi+3\,\xi^2+2\,\xi^3+\xi^4\right)u^5+O[u]^7$$

5. Distributions with at most three offspring

We recall that the basic definitions are given in Section 1

5.1. Important properties of the function $f_{F3}(x)$ and verification of Remarks 4.8 and 4.9

The following is (4.34) :

```
In[]:= fF3[x_] := φF3[p0, p2, p3][x] - φF3FL[p0, p2, p3][x]
```

Simple checks:

```
In[]:= Simplify[fF3[PinfF3[p0, p2, p3]]]
Out[]=
0

In[]:= Simplify[D[fF3[x], x] /. x → PinfF3[p0, p2, p3]]
Out[]=
0
```

Assumption: admissible region R in (4.33):

```
In[]:= assumeR = p0 > 0 && p2 ≥ 0 && p3 > 0 && p0 + p2 + p3 ≤ 1 && p0 < p2 + 2 p3;
```

Define p0plus, p0r, p0gamma in (4.36), (4.37), (4.38):

In[]:=

$$\text{p0plus} = \frac{\text{p3} - (\text{p2} + \text{p3})^2}{4\,\text{p3}};$$

$$\text{p0r} = \frac{1}{2} - \frac{(\text{p2} + \text{p3})\left(\text{p2} + \text{p3} + \sqrt{8\,\text{p3} + (\text{p2} + \text{p3})^2}\right)}{8\,\text{p3}};$$

$$\text{p0gam} = \frac{1}{2} - \frac{2\,(\text{p2} + \text{p3})\,\hat{}\,2 + (\text{p2} + 3\,\text{p3})\,\sqrt{8\,\text{p3} + (\text{p2} + 3\,\text{p3})^2} - (\text{p2} + 3\,\text{p3})^2}{8\,\text{p3}};$$

Remark 4.8

(a)

In[•]:=
```
Simplify[D[fF3[x], {x, 2}] /. x → PinfF3[p0, p2, p3]]
```

Out[•]=

$$\frac{\left(-p3+4\,p0\,p3+(p2+p3)^2\right)\left(p2+3\,p3-\sqrt{4\,p0\,p3+(p2+p3)^2}\right)}{p3}$$

The following confirms (8.13):

In[•]:=

$$\text{Simplify}\left[\text{Reduce}\left[\frac{\left(-p3+4\,p0\,p3+(p2+p3)^2\right)\left(p2+3\,p3-\sqrt{4\,p0\,p3+(p2+p3)^2}\right)}{p3}>0\,\&\&\; p0>0\,\&\&\,p2\geq 0\,\&\&\,p3>0\right],\ \text{Assumptions}\to \text{assumeR}\,\&\&\,p0>\text{p0plus}\right]$$

Out[•]=

True

In[•]:=

$$\text{Simplify}\left[\text{Reduce}\left[\frac{\left(-p3+4\,p0\,p3+(p2+p3)^2\right)\left(p2+3\,p3-\sqrt{4\,p0\,p3+(p2+p3)^2}\right)}{p3}>0\,\&\&\; p0>0\,\&\&\,p2\geq 0\,\&\&\,p3>0\right],\ \text{Assumptions}\to \text{assumeR}\,\&\&\,p0\leq\text{p0plus}\right]$$

Out[•]=

False

(b)

In[•]:=
```
r /. substprF3[p0, p2, p3]
```

Out[•]=

$$\frac{2\,p0\,\sqrt{4\,p0\,p3+(p2+p3)^2}}{p2+p3+(1+2\,p0)\,\sqrt{4\,p0\,p3+(p2+p3)^2}}$$

In[•]:=

$$\text{Simplify}\left[\text{Solve}\left[p0==\frac{2\,p0\,\sqrt{4\,p0\,p3+(p2+p3)^2}}{p2+p3+(1+2\,p0)\,\sqrt{4\,p0\,p3+(p2+p3)^2}},\ p0\right]\right]$$

Solve: There may be values of the parameters for which some or all solutions are not valid.

Out[•]=

$$\left\{\left\{p0\to-\frac{p2^2-4\,p3+2\,p2\,p3+p3^2+(p2+p3)\,\sqrt{p2^2+2\,p2\,p3+p3\,(8+p3)}}{8\,p3}\right\},\right.$$
$$\left.\left\{p0\to-\frac{p2^2-4\,p3+2\,p2\,p3+p3^2-(p2+p3)\,\sqrt{p2^2+2\,p2\,p3+p3\,(8+p3)}}{8\,p3}\right\},\ \{p0\to 0\}\right\}$$

In[•]:=
```
p0r
```

Out[•]=

$$\frac{1}{2}-\frac{(p2+p3)\left(p2+p3+\sqrt{8\,p3+(p2+p3)^2}\right)}{8\,p3}$$

In[•]:=

$$\text{Simplify}\left[p0r-\left(-\frac{p2^2-4\,p3+2\,p2\,p3+p3^2+(p2+p3)\,\sqrt{p2^2+2\,p2\,p3+p3\,(8+p3)}}{8\,p3}\right)\right]$$

Out[•]=

0

The other solution is not admissible (p0 = 0 isn't either)

In[∘]:=
```
Simplify[Reduce[1 > -(p2^2 - 4 p3 + 2 p2 p3 + p3^2 - (p2 + p3) Sqrt[p2^2 + 2 p2 p3 + p3 (8 + p3)])/(8 p3) > 0 &&
  -(p2^2 - 4 p3 + 2 p2 p3 + p3^2 - (p2 + p3) Sqrt[p2^2 + 2 p2 p3 + p3 (8 + p3)])/(8 p3) < p2 + 2 p3], Assumptions ->
  assumeR && -(p2^2 - 4 p3 + 2 p2 p3 + p3^2 - (p2 + p3) Sqrt[p2^2 + 2 p2 p3 + p3 (8 + p3)])/(8 p3) + p2 + p3 ≤ 1]
```

Out[∘]=
```
False
```

(c)

In[∘]:=
```
Simplify[Solve[gammaF3[p0, p2, p3] × meanF3[p0, p2, p3] == 1, p0]]
```

Solve: There may be values of the parameters for which some or all solutions are not valid.

Out[∘]=

$$\left\{\{p0 \to p2 + 2\,p3\},\ \left\{p0 \to -\frac{p2^2 - 4\,p3 - 2\,p2\,p3 - 7\,p3^2 + (p2 + 3\,p3)\sqrt{p2^2 + 6\,p2\,p3 + p3\,(8 + 9\,p3)}}{8\,p3}\right\},\ \left\{p0 \to \frac{-p2^2 + 4\,p3 + 2\,p2\,p3 + 7\,p3^2 + (p2 + 3\,p3)\sqrt{p2^2 + 6\,p2\,p3 + p3\,(8 + 9\,p3)}}{8\,p3}\right\}\right\}$$

In[∘]:=
```
Simplify[p0gam - (-(p2^2 - 4 p3 - 2 p2 p3 - 7 p3^2 + (p2 + 3 p3) Sqrt[p2^2 + 6 p2 p3 + p3 (8 + 9 p3)])/(8 p3))]
```

Out[∘]=
```
0
```

The other two solutions are not admissible:

In[∘]:=
```
Simplify[Reduce[(-p2^2 + 4 p3 + 2 p2 p3 + 7 p3^2 + (p2 + 3 p3) Sqrt[p2^2 + 6 p2 p3 + p3 (8 + 9 p3)])/(8 p3) > 0 &&
  (-p2^2 + 4 p3 + 2 p2 p3 + 7 p3^2 + (p2 + 3 p3) Sqrt[p2^2 + 6 p2 p3 + p3 (8 + 9 p3)])/(8 p3) <
   p2 + 2 p3], Assumptions -> assumeR]
```

Out[∘]=
```
False
```

Remark 4.9

(a) is obvious

(b)

In[∘]:=
```
Simplify[Reduce[p0r > 0, p2], Assumptions -> assumeR]
```

Out[∘]=

$$p2 + p3 < \sqrt{p3}$$

In[∘]:=
```
Simplify[Reduce[p0r > p0plus, p2], Assumptions -> assumeR]
```

Out[∘]=

$$p2 + p3 < \sqrt{p3}$$

In[∘]:= `Simplify[Reduce[p0r + p2 + p3 < 1 && 0 ≤ p2 < 1 && p2 + p3 < 1], Assumptions → assumeR]`

Out[∘]= True

In[∘]:= `Simplify[Reduce[p0r < p2 + 2 p3 && 0 ≤ p2 < 1 && p2 + p3 < 1], Assumptions → assumeR]`

Out[∘]= $6\,p3 > 1 \;||\; \sqrt{p3\,(4 + p3)} < 2\,p2 + 5\,p3$

(c)

In[∘]:= `Simplify[Reduce[0 < p0gam, p2], Assumptions → assumeR]`

Out[∘]= $\sqrt{p3\,(4 + p3)} > 2\,p2 + 3\,p3$

In[∘]:= `Simplify[Reduce[`$\sqrt{p3\,(4 + p3)}$` - 3 p3 > 0], Assumptions → assumeR]`

Out[∘]= $2\,p3 < 1$

In[∘]:= `Simplify[Reduce[p0gam < p0r], Assumptions → assumeR]`

Out[∘]= True

(d)

In[∘]:= `Simplify[Reduce[p0gam < p0plus && p2 ≥ 0], Assumptions → assumeR]`

Out[∘]= $9\,p3 > 1 \;||\; p2 + 3\,p3 > \sqrt{p3}$

In[∘]:= `Simplify[Reduce[p0plus < p2 + 2 p3 && p2 ≥ 0], Assumptions → assumeR]`

Out[∘]= $9\,p3 > 1 \;||\; p2 + 3\,p3 > \sqrt{p3}$

In[∘]:= `Simplify[Reduce[p0gam < p2 + 2 p3 && p2 ≥ 0], Assumptions → assumeR]`

Out[∘]= $9\,p3 > 1 \;||\; p2 + 3\,p3 > \sqrt{p3}$

(e)

In[∘]:= `Simplify[Reduce[`$\sqrt{p3} - 3\,p3 < \frac{1}{2}\left(\sqrt{p3\,(4 + p3)} - 5\,p3\right) < \frac{1}{2}\left(\sqrt{p3\,(4 + p3)} - 3\,p3\right) < \sqrt{p3} - p3$`],`
`Assumptions → assumeR]`

Out[∘]= True

5.2. Elements for the proof of Lemma 4.10

5.2.1 We show (C.2)

In[∘]:= `FullSimplify[Solve[`$\sqrt{4\,p0\,p3 + (p2 + p3)^2} == z$`, p0]]`

Solve: There may be values of the parameters for which some or all solutions are not valid.

Out[∘]= $\left\{\left\{p0 \to \frac{-(p2 + p3)^2 + z^2}{4\,p3}\right\}\right\}$

In[∘]:=
```
Factor[
 FullSimplify[(gammaF3[p0, p2, p3] × meanF3[p0, p2, p3] - 1 /. p0 → -(p2^2 + 2 p2 p3 + p3^2 - z^2)/(4 p3)),
  Assumptions → z > 0]]
```

Out[∘]=

$$-\frac{(p2+3\,p3-z)^2\,\left(-2\,p3+p2\,z+3\,p3\,z+z^2\right)}{8\,p3^2}$$

In[∘]:=
```
FullSimplify[-((p2 + 3 p3 - z)^2 (-2 p3 + p2 z + 3 p3 z + z^2))/(8 p3^2) /. z → Sqrt[4 p0 p3 + (p2 + p3)^2]]
```

Out[∘]=

$$-\frac{1}{8\,p3^2}\left(p2+3\,p3-\sqrt{4\,p0\,p3+(p2+p3)^2}\right)^2\left(-2\,p3+4\,p0\,p3+(p2+p3)^2+p2\,\sqrt{4\,p0\,p3+(p2+p3)^2}+3\,p3\,\sqrt{4\,p0\,p3+(p2+p3)^2}\right)$$

Define:

In[∘]:=
```
facgam = -(-2 p3 + 4 p0 p3 + (p2 + p3)^2 + p2 Sqrt[4 p0 p3 + (p2 + p3)^2] + 3 p3 Sqrt[4 p0 p3 + (p2 + p3)^2]);
```

In[∘]:=
```
Simplify[Solve[facgam == 0, p0], Assumptions → p2 ≥ 0 && p3 ≥ 0]
```

Solve: There may be values of the parameters for which some or all solutions are not valid.

Out[∘]=

$$\left\{\left\{p0\to-\frac{p2^2-4\,p3-2\,p2\,p3-7\,p3^2+(p2+3\,p3)\,\sqrt{p2^2+6\,p2\,p3+p3\,(8+9\,p3)}}{8\,p3}\right\},\left\{p0\to\frac{-p2^2+4\,p3+2\,p2\,p3+7\,p3^2+(p2+3\,p3)\,\sqrt{p2^2+6\,p2\,p3+p3\,(8+9\,p3)}}{8\,p3}\right\}\right\}$$

Therefore, p0r is indeed a zero of facgam (the only on in R)

5.2.2 We check (C.3a) and (C.3b)

In[∘]:=
```
FullSimplify[Reduce[facgam > 0 && p2 ≥ 0 && p3 > 0], Assumptions → assumeR && p0 < p0gam]
```

Out[∘]=

```
True
```

In[∘]:=
```
FullSimplify[Reduce[facgam > 0 && p0 ≥ p0gam && p2 ≥ 0 && p3 > 0], Assumptions → assumeR]
```

Out[∘]=

```
False
```

In[∘]:=
```
FullSimplify[Reduce[facgam < 0 && p2 ≥ 0 && p3 > 0], Assumptions → assumeR && p0 > p0gam]
```

Out[∘]=

```
True
```

In[∘]:=
```
FullSimplify[Reduce[facgam < 0 && p0 ≤ p0gam && p2 ≥ 0 && p3 > 0], Assumptions → assumeR]
```

Out[∘]=

```
False
```

The rest of the proof of Lemma 4.10 is clear from Appendix C.

5.3. Elements for the proof of Theorem 4.11

We check (C.4), which was obtained by the substitution

In[•]:=
```
Simplify[Solve[PinfF3[p0, p2, p3] == PiF3, p0]]
```

Solve: There may be values of the parameters for which some or all solutions are not valid.

Out[•]=
```
{{p0 → PiF3 (p2 + p3 + p3 PiF3)}}
```

Define

In[•]:=
```
fF3Pinf[x_] := (1 - x) (PinfF3[p0, p2, p3] - x)^2
   ((-p3 + (p2 + p3 + 2 p3 PinfF3[p0, p2, p3]) (p2 + p3 + p3 PinfF3[p0, p2, p3] + p3 x)) /
     (1 + (p2 + p3 + 2 p3 PinfF3[p0, p2, p3]) (PinfF3[p0, p2, p3] - x)));
```

The following confirms (C.4):

In[•]:=
```
Simplify[fF3[x] - fF3Pinf[x]]
```

Out[•]=
```
0
```

Remark C.1 (b): we check fF3''' if p0=p0plus

In[•]:=
```
Simplify[D[fF3Pinf[x], {x, 3}] /. x → PinfF3[p0, p2, p3] /. p0 → p0plus]
```

Out[•]=

$$3\sqrt{p3}\left(p2-\sqrt{p3}+3\,p3\right)$$

No further tedious calculations are required to follow the proof of Theorem 4.12 in Appendix C.

5.4. Code for figures such as Figure 4.2A

In[•]:=
```
PinfF3[p0, p2, p3] /. {p3 → p2 / 2, p0 → p2} /. p2 → 0.15
```

Out[•]=
```
0.561553
```

In[•]:=
```
Plot[fF3[x] /. {p3 → p2 / 2, p0 → p2} /. p2 → 0.15, {x, 0, 1},
 AxesLabel → {"x", "f(x)"}, LabelStyle → Directive[Black, FontSize → 11]]
```

Out[•]=

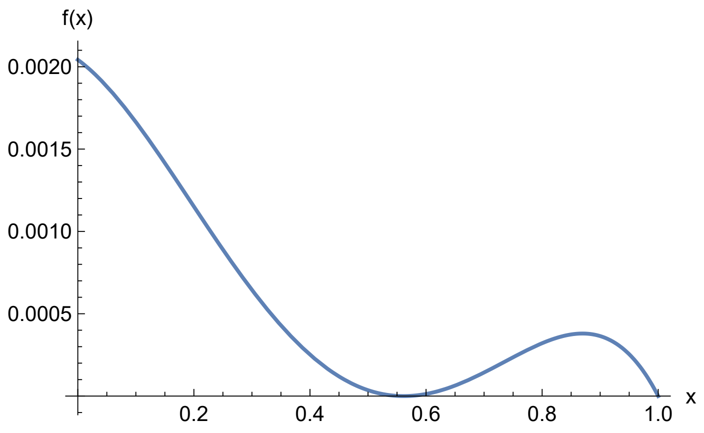

5.5. Code for figures such as Figure 4.3A

In[∘]:=

```
p0rplot[p2_, p3_] := 1/2 - ((p2 + p3) (p2 + p3 + Sqrt[8 p3 + (p2 + p3)^2]))/(8 p3);
p0plusplot[p2_, p3_] := (p3 - (p2 + p3)^2)/(4 p3);
```

$$p0rplot[p2\_, p3\_] := \frac{1}{2} - \frac{(p2 + p3)\left(p2 + p3 + \sqrt{8\,p3 + (p2 + p3)^2}\right)}{8\,p3};$$

$$p0plusplot[p2\_, p3\_] := \frac{p3 - (p2 + p3)^2}{4\,p3};$$

In[∘]:=

```
plotR1 = RegionPlot3D[p0rplot[p2, p3] ≤ p0 ≤ p2 + 2 p3 && p0 + p2 + p3 < 1,
  {p0, 0, 2 / 3}, {p2, 0, 1}, {p3, 0.00001, 1},
  AxesLabel → {Style["p0", FontFamily → "Helvetica", FontSize → 14, Black],
    Style["p2", FontFamily → "Helvetica", FontSize → 14, Black],
    Style["p3 ", FontFamily → "Helvetica", FontSize → 14, Black]},
  Ticks → {{0, {0.333, "1/3"}, {0.667, "2/3"}}, {0, {0.333, "1/3"}, {0.667, "2/3"}, 1},
    {0, {0.333, "1/3"}, {0.667, "2/3"}, 1}},
  TicksStyle → Directive[FontFamily → "Helvetica", FontSize → 12, Black],
  LabelStyle → Directive[FontFamily → "Helvetica", FontSize → 12, Black],
  PlotPoints → 100, PlotStyle → Yellow, Mesh → 10]
```

Out[∘]=

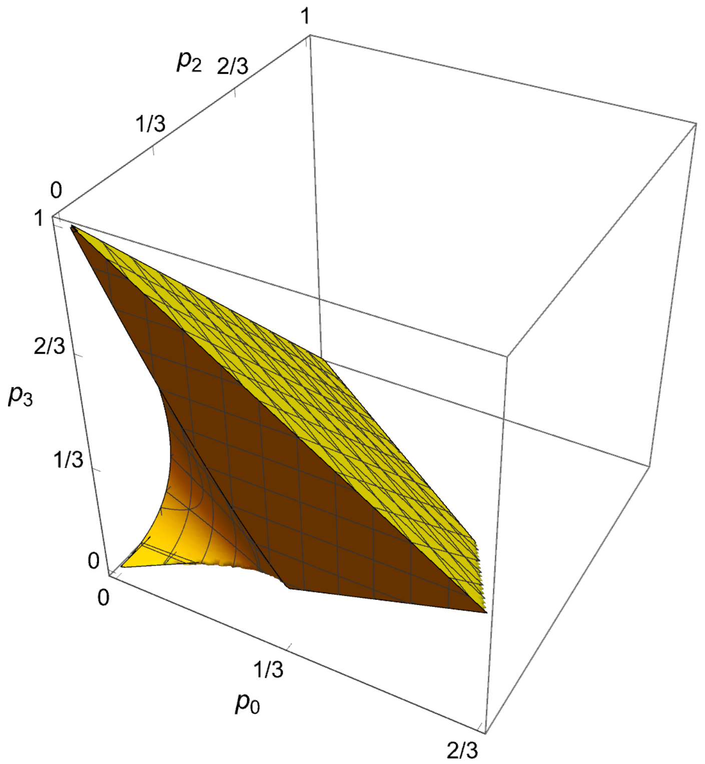

In[ ]:=
```
plotR2 = RegionPlot3D[p0gam < p0 ≤ p0rplot[p2, p3] && 0 < p0 < p2 + 2 p3,
  {p0, 0, 0.4}, {p2, 0, 0.3}, {p3, 0.00001, 1},
  AxesLabel → {p0, p2, p3}, PlotPoints → 100, PlotStyle → Red, Mesh → 10]
```

Out[ ]=

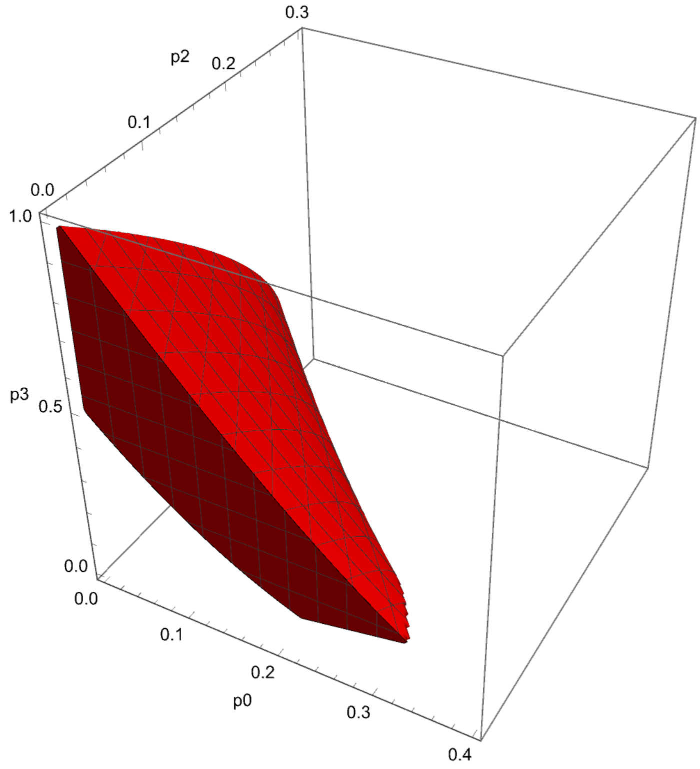

In[ ]:=
```
plotR3 = RegionPlot3D[0 < p0 ≤ p0gam && p0 < p2 + 2 p3,
  {p0, 0, 0.25}, {p2, 0, 0.2}, {p3, 0.00001, 0.5},
  AxesLabel → {p0, p2, p3}, PlotPoints → 100, PlotStyle → Green, Mesh → 10]
```

Out[ ]=

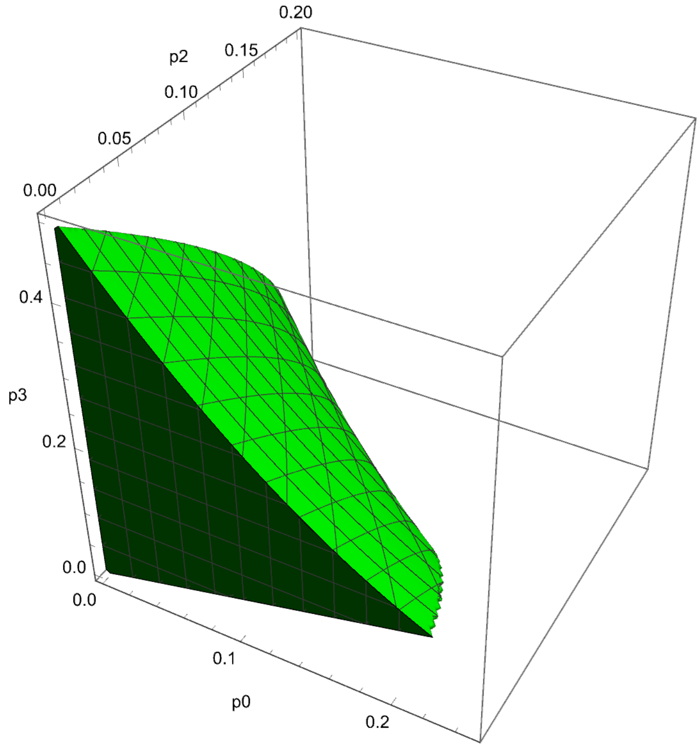

In[ ]:=
```
Show[plotR1, plotR2, plotR3, ViewPoint → {2.2, -1.9, 0.8},
 AxesEdge → {{0, 0}, Automatic, {0, 0}}]
```

Out[ ]=

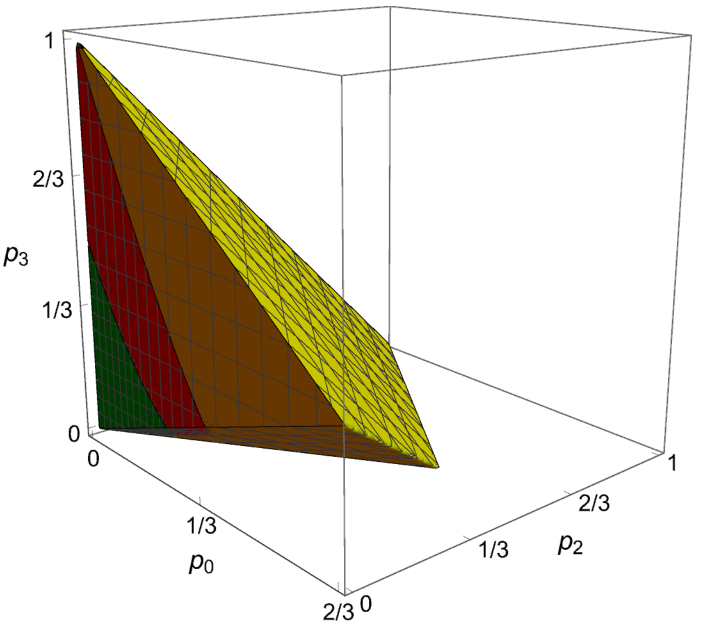

6. Bounds and approximations for the ultimate survival probability S_φ^∞ (Sects. 5.1, 5.2, 5.3, 5.5, 5.6, 5.7 in the ms)

This section contains most of the tedious calculations needed for the results in Section 5 of the ms .

6.1. General definitions of the bounds

We provide the definitions for S_φ^∞, whereas in the original papers they were defined for P_φ^∞.
The notation is very similar to that in the manuscript and should be self-explanatory

6.1.1 Quine’s (1976) lower and upper bounds for S_φ^∞

In[ ]:=

$$\phi\text{bound} = \frac{2\,(m-1)}{b};$$

$$\text{LQbound} = \frac{2\,(m-1)}{b} + \left(\frac{2\,(m-1)}{b}\right)\hat{}\,2\ \frac{1}{3\,b}\ \varphi^{(3,0)}\left[\frac{4+b-4\,m}{b},\ s\right];$$

$$\text{UQbound} = \phi\text{bound} + \phi\text{bound}\,\hat{}\,2\ \frac{c}{3\,b}\left(1-\frac{4\,c}{3\,b}\ \phi\text{bound}\right)\hat{}\,(-3/2);$$

6.1.2 Daley and Narayan's (1980) upper bound for S_φ^∞

In[ ]:=

$$\text{UDNbound} = \frac{3\,b-\sqrt{9\,b^2-24\,c\,(m-1)}}{2\,c};$$

D & N gave this bound in the equivalent form $\frac{4\ (m-1)\ /\ b}{1+\sqrt{1-8\ (m-1)\ c\ /\ (3\ b\text{^}2)}}$:

In[•]:=
```
FullSimplify[UDNbound - (4 (m - 1) / b)/(1 + Sqrt[1 - 8 (m - 1) c / (3 b^2)]), Assumptions → b > 0]
```

Out[•]=
```
0
```

Remark: The UDNbound is derived by solving fLNB[x] = x with k = 2 (see From 2007, who refers to Narayan 1981), where

In[•]:=
```
fLNB[x_] := 1 - m + b/k - (c - (k - 2) b)/(k (k + 1)) +
  x (m - b/(k - 1) + (c - (k - 2) b)/(k (k - 1))) + x^k ((k - 1) b - c)/(k (k - 1)) + x^(k + 1) (c - (k - 2) b)/(k (k + 1))
```

is a lower bound to the generating function φ (in general, k = IntegerPart[1 + b/m]) . Note that in From' s eq (1.13) there are typos! Verification:

In[•]:=
```
Simplify[Solve[(fLNB[x] /. k → 2) == x, x]]
```

Out[•]=

$$\left\{\{x\to 1\},\ \left\{x\to -\frac{3\,b-2\,c+\sqrt{9\,b^2-24\,c\,(-1+m)}}{2\,c}\right\},\ \left\{x\to \frac{-3\,b+2\,c+\sqrt{9\,b^2-24\,c\,(-1+m)}}{2\,c}\right\}\right\}$$

6.2. Series expansions of S_φ^∞ and γ_φ for general φ

We set Pinf = Sum[δ[i] (-s)^i, {i, 0, 4}] with δ[0] = 1

In[•]:=
```
Clear[δ]
```

In[•]:=
```
δ[0] = 1;
δ[1] = θ;
Pinfser = Sum[δ[i] (-s)^i, {i, 0, 4}];
```

We use the following assumptions and substitutions:

In[•]:=
```
φ[1, s_] := 1;
φ^(1,0)[1, s_] := 1 + s;
μ[0, k_] := 0;
μ[1, 1] = 1;
μ[1, 2] = 0;
μ[1, 3] = 0;
μ[1, 4] = 0;
substderiv = {φ^(k_,1_)[1, 0] → μ[k, 1]};
```

In[•]:=
```
Simplify[Series[φ[Pinfser, s] - Pinfser, {s, 0, 5}] //. substderiv] // Normal
```

Out[•]=

$$\frac{1}{2} s^2 \theta\, (-2+\theta\,\mu[2,0]) + s^3 \left(\delta[2]\,(1-\theta\,\mu[2,0]) - \frac{1}{6}\theta^2\,(-3\,\mu[2,1]+\theta\,\mu[3,0])\right) + s^4 \left(\delta[3]\,(-1+\theta\,\mu[2,0]) + \frac{1}{24}\left(12\,\delta[2]^2\,\mu[2,0] + 12\,\theta\,\delta[2]\,(-2\,\mu[2,1]+\theta\,\mu[3,0]) + \theta^2\left(6\,\mu[2,2]-4\,\theta\,\mu[3,1]+\theta^2\,\mu[4,0]\right)\right)\right) + s^5 \left(\delta[4]\,(1-\theta\,\mu[2,0]) + \frac{1}{120}\left(60\,\delta[2]^2\,(\mu[2,1]-\theta\,\mu[3,0]) - 20\,\delta[2]\left(6\,\delta[3]\times\mu[2,0] + \theta\left(3\,\mu[2,2]-3\,\theta\,\mu[3,1]+\theta^2\,\mu[4,0]\right)\right) + \theta\left(60\,\delta[3]\,(2\,\mu[2,1]-\theta\,\mu[3,0]) + \theta\left(10\,\mu[2,3]-10\,\theta\,\mu[3,2]+5\,\theta^2\,\mu[4,1]-\theta^3\,\mu[5,0]\right)\right)\right)\right)$$

In[•]:=
```
coeffs = CoefficientList[%, s]
```

Out[•]=

$$\left\{0,\ 0,\ \frac{1}{2}\theta\,(-2+\theta\,\mu[2,0]),\ \delta[2]\,(1-\theta\,\mu[2,0]) - \frac{1}{6}\theta^2\,(-3\,\mu[2,1]+\theta\,\mu[3,0]),\ \delta[3]\,(-1+\theta\,\mu[2,0]) + \frac{1}{24}\left(12\,\delta[2]^2\,\mu[2,0] + 12\,\theta\,\delta[2]\,(-2\,\mu[2,1]+\theta\,\mu[3,0]) + \theta^2\left(6\,\mu[2,2]-4\,\theta\,\mu[3,1]+\theta^2\,\mu[4,0]\right)\right),\ \delta[4]\,(1-\theta\,\mu[2,0]) + \frac{1}{120}\left(60\,\delta[2]^2\,(\mu[2,1]-\theta\,\mu[3,0]) - 20\,\delta[2]\left(6\,\delta[3]\times\mu[2,0]+\theta\left(3\,\mu[2,2]-3\,\theta\,\mu[3,1]+\theta^2\,\mu[4,0]\right)\right) + \theta\left(60\,\delta[3]\,(2\,\mu[2,1]-\theta\,\mu[3,0]) + \theta\left(10\,\mu[2,3]-10\,\theta\,\mu[3,2]+5\,\theta^2\,\mu[4,1]-\theta^3\,\mu[5,0]\right)\right)\right)\right\}$$

The non-trivial (second) solution below gives the desired coefficients; see eqs (5.9) -- (5.11) in the ms:

In[•]:=
```
Simplify[Solve[Delete[coeffs, {{1}, {2}}] == 0, Table[δ[i], {i, 1, 4}]]]
```

Out[•]=

$$\left\{\{\theta\to 0,\ \delta[2]\to 0,\ \delta[3]\to 0,\ \delta[4]\to 0\},\ \left\{\theta\to\frac{2}{\mu[2,0]},\ \delta[2]\to\frac{6\,\mu[2,0]\times\mu[2,1]-4\,\mu[3,0]}{3\,\mu[2,0]^3},\ \delta[3]\to\frac{1}{9\,\mu[2,0]^5}\left(-9\,\mu[2,0]^3\,\mu[2,2]+16\,\mu[3,0]^2+6\,\mu[2,0]^2\left(3\,\mu[2,1]^2+2\,\mu[3,1]\right)-6\,\mu[2,0]\,(6\,\mu[2,1]\times\mu[3,0]+\mu[4,0])\right),\ \delta[4]\to\frac{1}{135\,\mu[2,0]^7}\left(45\,\mu[2,0]^5\,\mu[2,3]-400\,\mu[3,0]^3-90\,\mu[2,0]^4\,(3\,\mu[2,1]\times\mu[2,2]+\mu[3,2])+300\,\mu[2,0]\times\mu[3,0]\,(4\,\mu[2,1]\times\mu[3,0]+\mu[4,0])+90\,\mu[2,0]^3\left(3\,\mu[2,1]^3+3\,\mu[2,2]\times\mu[3,0]+6\,\mu[2,1]\times\mu[3,1]+\mu[4,1]\right)-12\,\mu[2,0]^2\left(90\,\mu[2,1]^2\,\mu[3,0]+40\,\mu[3,0]\times\mu[3,1]+30\,\mu[2,1]\times\mu[4,0]+3\,\mu[5,0]\right)\right)\right\}\right\}$$

In[•]:=
```
Expand[-9 μ[2, 0]^3 μ[2, 2] + 16 μ[3, 0]^2 +
  6 μ[2, 0]^2 (3 μ[2, 1]^2 + 2 μ[3, 1]) - 6 μ[2, 0] (6 μ[2, 1] × μ[3, 0] + μ[4, 0])]
```

Out[•]=

$$18\,\mu[2,0]^2\,\mu[2,1]^2 - 9\,\mu[2,0]^3\,\mu[2,2] - 36\,\mu[2,0]\times\mu[2,1]\times\mu[3,0] + 16\,\mu[3,0]^2 + 12\,\mu[2,0]^2\,\mu[3,1] - 6\,\mu[2,0]\times\mu[4,0]$$

In[•]:=
```
δlist = {δ[2] → (6 μ[2, 0] × μ[2, 1] - 4 μ[3, 0])/(3 μ[2, 0]^3), δ[3] →
   1/(9 μ[2, 0]^5) (18 μ[2, 0]^2 μ[2, 1]^2 - 9 μ[2, 0]^3 μ[2, 2] - 36 μ[2, 0] × μ[2, 1] × μ[3, 0] +
      16 μ[3, 0]^2 + 12 μ[2, 0]^2 μ[3, 1] - 6 μ[2, 0] × μ[4, 0]), δ[4] → 1/(135 μ[2, 0]^7)
   (45 μ[2, 0]^5 μ[2, 3] - 400 μ[3, 0]^3 - 90 μ[2, 0]^4 (3 μ[2, 1] × μ[2, 2] + μ[3, 2]) +
      300 μ[2, 0] × μ[3, 0] (4 μ[2, 1] × μ[3, 0] + μ[4, 0]) + 90 μ[2, 0]^3
       (3 μ[2, 1]^3 + 3 μ[2, 2] × μ[3, 0] + 6 μ[2, 1] × μ[3, 1] + μ[4, 1]) - 12 μ[2, 0]^2
       (90 μ[2, 1]^2 μ[3, 0] + 40 μ[3, 0] × μ[3, 1] + 30 μ[2, 1] × μ[4, 0] + 3 μ[5, 0]))};
```

Now we compute the series expansion of gamma in (5.12) and (5.13):

In[•]:=
```
Simplify[Series[D[φ[x, s], x] /. x → 1 - θ s + δ[2] s^2 - δ[3] s^3 + δ[4] s^4, {s, 0, 4}] /.
    substderiv /. δlist /. θ → 2 / μ[2, 0]]
```

Out[•]=

$$1 - s + \frac{2\,\mu[3,0]\,s^2}{3\,\mu[2,0]^2} + \frac{\left(8\,\mu[3,0]^2 + 6\,\mu[2,0]^2\,\mu[3,1] - 6\,\mu[2,0]\,(2\,\mu[2,1]\times\mu[3,0] + \mu[4,0])\right)s^3}{9\,\mu[2,0]^4} + \frac{1}{135\,\mu[2,0]^6}\Big(200\,\mu[3,0]^3 + 45\,\mu[2,0]^4\,\mu[3,2] - 240\,\mu[2,0]\times\mu[3,0]\,(2\,\mu[2,1]\times\mu[3,0] + \mu[4,0]) - 90\,\mu[2,0]^3\,(\mu[2,2]\times\mu[3,0] + 2\,\mu[2,1]\times\mu[3,1] + \mu[4,1]) + 6\,\mu[2,0]^2\left(45\,\mu[2,1]^2\,\mu[3,0] + 40\,\mu[3,0]\times\mu[3,1] + 45\,\mu[2,1]\times\mu[4,0] + 9\,\mu[5,0]\right)\Big)\,s^4 + O[s]^5$$

In[•]:=
```
Expand[-1/2 (8 μ[3, 0]^2 + 6 μ[2, 0]^2 μ[3, 1] - 6 μ[2, 0] (2 μ[2, 1] × μ[3, 0] + μ[4, 0]))]
```

Out[•]=

$$6\,\mu[2,0]\times\mu[2,1]\times\mu[3,0] - 4\,\mu[3,0]^2 - 3\,\mu[2,0]^2\,\mu[3,1] + 3\,\mu[2,0]\times\mu[4,0]$$

We define

In[•]:=
```
γ[0] = 1;
γ[1] = 1;
γlist = {γ[2] → (2 μ[3, 0])/(3 μ[2, 0]^2), γ[3] → 2/(9 μ[2, 0]^4) (6 μ[2, 0] × μ[2, 1] × μ[3, 0] - 4 μ[3, 0]^2 -
      3 μ[2, 0]^2 μ[3, 1] + 3 μ[2, 0] × μ[4, 0]) , γ[4] → 1/(135 μ[2, 0]^6) (200 μ[3, 0]^3 +
      45 μ[2, 0]^4 μ[3, 2] - 240 μ[2, 0] × μ[3, 0] (2 μ[2, 1] × μ[3, 0] + μ[4, 0]) -
      90 μ[2, 0]^3 (μ[2, 2] × μ[3, 0] + 2 μ[2, 1] × μ[3, 1] + μ[4, 1]) + 6 μ[2, 0]^2
       (45 μ[2, 1]^2 μ[3, 0] + 40 μ[3, 0] × μ[3, 1] + 45 μ[2, 1] × μ[4, 0] + 9 μ[5, 0]))};
```

In[•]:=
```
Sinfser[s_, n_] := Sum[δ[k] (-1) ^ (k - 1) s^k, {k, 1, n}];
gammaser[s_, n_] := Sum[γ[k] (-s) ^k, {k, 0, n}]
```

6.3. Series expansions of Quine's and Daley and Narayana bounds

In[•]:=
```
Simplify[
 Series[ϕbound /. {m → 1 + s, b → φ^(2,0)[1, s], c → φ^(3,0)[1, s]}, {s, 0, 3}] /. substderiv]
```

Out[•]=

$$\frac{2\,s}{\mu[2,0]} - \frac{2\,\mu[2,1]\,s^2}{\mu[2,0]^2} + \frac{\left(2\,\mu[2,1]^2 - \mu[2,0]\times\mu[2,2]\right)s^3}{\mu[2,0]^3} + O[s]^4$$

In[•]:=
```
Simplify[Series[UQbound /. {m → 1 + s, b → φ^(2,0)[1, s], c → φ^(3,0)[1, s]}, {s, 0, 3}] /.
   substderiv] // Normal
```

Out[•]=

$$\frac{2\,s}{\mu[2,0]} + \frac{s^2\left(-6\,\mu[2,0]\times\mu[2,1] + 4\,\mu[3,0]\right)}{3\,\mu[2,0]^3} + \frac{1}{3\,\mu[2,0]^5}\,s^3\left(-3\,\mu[2,0]^3\,\mu[2,2] - 12\,\mu[2,0]\times\mu[2,1]\times\mu[3,0] + 16\,\mu[3,0]^2 + \mu[2,0]^2\left(6\,\mu[2,1]^2 + 4\,\mu[3,1]\right)\right)$$

In[•]:=
```
Simplify[Series[% - Sinfser[s, 3], {s, 0, 3}] /. δlist /. θ → 2 / μ[2, 0]]
```

Out[•]=

$$\frac{\left(32\,\mu[3,0]^2 + 6\,\mu[2,0]\times\mu[4,0]\right)s^3}{9\,\mu[2,0]^5} + O[s]^4$$

In[•]:=
```
Simplify[Series[LQbound /. {m → 1 + s, b → φ^(2,0)[1, s], c → φ^(3,0)[1, s]}, {s, 0, 3}] /.
   substderiv] // Normal
```

Out[•]=

$$\frac{2\,s}{\mu[2,0]} + \frac{s^2\left(-6\,\mu[2,0]\times\mu[2,1] + 4\,\mu[3,0]\right)}{3\,\mu[2,0]^3} + \frac{1}{3\,\mu[2,0]^4}\,s^3\left(-3\,\mu[2,0]^2\,\mu[2,2] + \mu[2,0]\left(6\,\mu[2,1]^2 + 4\,\mu[3,1]\right) - 4\left(3\,\mu[2,1]\times\mu[3,0] + 4\,\mu[4,0]\right)\right)$$

In[•]:=
```
Simplify[Series[% - Sinfser[s, 3], {s, 0, 3}] /. δlist /. θ → 2 / μ[2, 0]]
```

Out[•]=

$$-\frac{2\left(8\,\mu[3,0]^2 + 21\,\mu[2,0]\times\mu[4,0]\right)s^3}{9\,\mu[2,0]^5} + O[s]^4$$

In[•]:=
```
Simplify[Series[UDNbound /. {m → 1 + s, b → φ^(2,0)[1, s], c → φ^(3,0)[1, s]}, {s, 0, 3}] /.
   substderiv, Assumptions → μ[2, 0] > 0] // Normal
```

Out[•]=

$$\frac{2\,s}{\mu[2,0]} + \frac{2\,s^2\left(-3\,\mu[2,0]\times\mu[2,1] + 2\,\mu[3,0]\right)}{3\,\mu[2,0]^3} + \frac{1}{9\,\mu[2,0]^5}\,s^3\left(-9\,\mu[2,0]^3\,\mu[2,2] - 36\,\mu[2,0]\times\mu[2,1]\times\mu[3,0] + 16\,\mu[3,0]^2 + 6\,\mu[2,0]^2\left(3\,\mu[2,1]^2 + 2\,\mu[3,1]\right)\right)$$

In[•]:=
```
Simplify[Series[% - Sinfser[s, 3], {s, 0, 3}] /. δlist /. θ → 2 / μ[2, 0]]
```

Out[•]=

$$\frac{2\,\mu[4,0]\,s^3}{3\,\mu[2,0]^4} + O[s]^4$$

Therefore, the series expansions of the UQbound, LQbound, and UDNbound have correct terms of

order 1 and 2 in s

6.4. Series expansions for specific distributions (Sects. 5.5 - 5.7)

Poisson distribution (Sect. 5.5)

The Lambert function has the following Taylor expansion about x=0: $W(x) = \sum_{k=1}^{\infty} \frac{(-k)^{k-1}}{k!} x^k$.

In[ ]:= $\text{Table}\left[\frac{(-k)^{k-1}}{k!} x^k, \{k, 1, 7\}\right]$

Out[ ]=

$$\left\{x, -x^2, \frac{3 x^3}{2}, -\frac{8 x^4}{3}, \frac{125 x^5}{24}, -\frac{54 x^6}{5}, \frac{16807 x^7}{720}\right\}$$

Here, we can work with the explicit expression for SinfPoi

```
In[ ]:= Simplify[Series[ProductLog[x], {x, 0, 7}], Assumptions → x > 0]
```

Out[ ]=

$$x - x^2 + \frac{3 x^3}{2} - \frac{8 x^4}{3} + \frac{125 x^5}{24} - \frac{54 x^6}{5} + \frac{16807 x^7}{720} + O[x]^8$$

```
In[ ]:= Simplify[Series[SinfPoi[1 + s], {s, 0, 4}], Assumptions → s > 0]
```

Out[ ]=

$$2 s - \frac{8 s^2}{3} + \frac{28 s^3}{9} - \frac{464 s^4}{135} + O[s]^5$$

```
In[ ]:= Simplify[Series[gammaPoi[1 + s], {s, 0, 4}], Assumptions → s > 0]
```

Out[ ]=

$$1 - s + \frac{2 s^2}{3} - \frac{4 s^3}{9} + \frac{44 s^4}{135} + O[s]^5$$

Binomial distribution (Sect. 5.6)

In[ ]:= $\text{Table}\left[\mu[k, l] \to D\left[D\left[\varphi\text{Bin}\left[n, \frac{1+s}{n}\right][x], \{x, k\}\right], \{s, l\}\right] \text{ /. } \{x \to 1, s \to 0\}, \{k, 1, 5\}, \{l, 0, 5 - k\}\right] \text{ // Flatten}$

Out[ ]=

$$\left\{\mu[1, 0] \to 1, 1 \to 1, 0 \to 0, 0 \to 0, 0 \to 0, \mu[2, 0] \to \frac{-1 + n}{n}, \mu[2, 1] \to \frac{2 (-1 + n)}{n}, \mu[2, 2] \to \frac{2 (-1 + n)}{n}, \mu[2, 3] \to 0, \mu[3, 0] \to \frac{(-2 + n) (-1 + n)}{n^2}, \mu[3, 1] \to \frac{3 (-2 + n) (-1 + n)}{n^2}, \mu[3, 2] \to \frac{6 (-2 + n) (-1 + n)}{n^2}, \mu[4, 0] \to \frac{(-3 + n) (-2 + n) (-1 + n)}{n^3}, \mu[4, 1] \to \frac{4 (-3 + n) (-2 + n) (-1 + n)}{n^3}, \mu[5, 0] \to \frac{(-4 + n) (-3 + n) (-2 + n) (-1 + n)}{n^4}\right\}$$

In[∘]:=
```
substμBin = {θ → 2 n/(n - 1), μ[1, 0] → 1, 1 → 1, 0 → 0, 0 → 0,
   0 → 0, μ[2, 0] → (-1 + n)/n, μ[2, 1] → 2 (-1 + n)/n, μ[2, 2] → 2 (-1 + n)/n,
   μ[2, 3] → 0, μ[3, 0] → (-2 + n) (-1 + n)/n^2, μ[3, 1] → 3 (-2 + n) (-1 + n)/n^2,
   μ[3, 2] → 6 (-2 + n) (-1 + n)/n^2, μ[4, 0] → (-3 + n) (-2 + n) (-1 + n)/n^3,
   μ[4, 1] → 4 (-3 + n) (-2 + n) (-1 + n)/n^3, μ[5, 0] → (-4 + n) (-3 + n) (-2 + n) (-1 + n)/n^4};
```

In[∘]:= `Simplify[Series[Sinfser[s, 4] /. δlist /. substμBin, {s, 0, 4}]]`

Out[∘]=

$$\frac{2\,n\,s}{-1+n}-\frac{4\,(n\,(-1+2\,n))\,s^2}{3\,(-1+n)^2}+\frac{2\,n\,\left(5-17\,n+14\,n^2\right)\,s^3}{9\,(-1+n)^3}-\frac{8\,\left(n\,\left(-17+78\,n-117\,n^2+58\,n^3\right)\right)\,s^4}{135\,(-1+n)^4}+O[s]^5$$

In[∘]:=
```
SinfBinser[s_, 4] :=
  2 n/(n - 1) s - 4 (n (-1 + 2 n)) s^2/(3 (-1 + n)^2) + 2 n (5 - 17 n + 14 n^2) s^3/(9 (-1 + n)^3) - 8 n (-17 + 78 n - 117 n^2 + 58 n^3) s^4/(135 (-1 + n)^4)
```

In[∘]:= `FullSimplify[Series[gammaser[s, 4] /. δlist /. γlist /. substμBin, {s, 0, 4}]]`

Out[∘]=

$$1-s+\frac{2\,(-2+n)\,s^2}{3\,(-1+n)}-\frac{4\,(-2+n)^2\,s^3}{9\,(-1+n)^2}+\frac{2\,(-2+n)\,(79+n\,(-79+22\,n))\,s^4}{135\,(-1+n)^3}+O[s]^5$$

Other version:

In[∘]:=
```
FullSimplify[
  Series[n p PinfB/((1 - p + p PinfB)) /. p → (1 + s)/n /. PinfB → 1 - SinfBinser[s, 4], {s, 0, 4}]]
```

Out[∘]=

$$1-s+\frac{2\,(-2+n)\,s^2}{3\,(-1+n)}-\frac{4\,(-2+n)^2\,s^3}{9\,(-1+n)^2}+\frac{2\,(-2+n)\,(79+n\,(-79+22\,n))\,s^4}{135\,(-1+n)^3}+O[s]^5$$

Negative binomial distribution (Sect. 5.7)

In[∘]:=
```
Simplify[Table[μ[k, l] → D[D[φNB[n, n/(n + 1 + s)][x], {x, k}], {s, l}] /. {x → 1, s → 0},
   {k, 1, 5}, {l, 0, 5 - k}] // Flatten]
```

Out[∘]=

$$\left\{\mu[1,0]\to 1,\ 1\to 1,\ 0\to 0,\ 0\to 0,\ 0\to 0,\ \mu[2,0]\to 1+\frac{1}{n},\ \mu[2,1]\to 2+\frac{2}{n},\ \mu[2,2]\to 2+\frac{2}{n},\ \mu[2,3]\to 0,\ \mu[3,0]\to\frac{(1+n)\,(2+n)}{n^2},\ \mu[3,1]\to\frac{3\,(1+n)\,(2+n)}{n^2},\ \mu[3,2]\to\frac{6\,(1+n)\,(2+n)}{n^2},\ \mu[4,0]\to\frac{(1+n)\,(2+n)\,(3+n)}{n^3},\ \mu[4,1]\to\frac{4\,(1+n)\,(2+n)\,(3+n)}{n^3},\ \mu[5,0]\to\frac{(1+n)\,(2+n)\,(3+n)\,(4+n)}{n^4}\right\}$$

In[•]:=
```
substμNB = {θ → 2n/(1+n), μ[1, 0] → 1, 1 → 1, 0 → 0, 0 → 0, 0 → 0, μ[2, 0] → 1 + 1/n,
   μ[2, 1] → 2 + 2/n, μ[2, 2] → 2 + 2/n, μ[2, 3] → 0, μ[3, 0] → ((1+n) (2+n))/n^2,
   μ[3, 1] → (3 (1+n) (2+n))/n^2, μ[3, 2] → (6 (1+n) (2+n))/n^2, μ[4, 0] → ((1+n) (2+n) (3+n))/n^3,
   μ[4, 1] → (4 (1+n) (2+n) (3+n))/n^3, μ[5, 0] → ((1+n) (2+n) (3+n) (4+n))/n^4};
```

$$\mathrm{subst}\mu\mathrm{NB} = \left\{\theta \to \frac{2n}{1+n},\ \mu[1,0]\to 1,\ 1\to 1,\ 0\to 0,\ 0\to 0,\ 0\to 0,\ \mu[2,0]\to 1+\frac{1}{n},\ \mu[2,1]\to 2+\frac{2}{n},\ \mu[2,2]\to 2+\frac{2}{n},\ \mu[2,3]\to 0,\ \mu[3,0]\to \frac{(1+n)(2+n)}{n^2},\ \mu[3,1]\to \frac{3(1+n)(2+n)}{n^2},\ \mu[3,2]\to \frac{6(1+n)(2+n)}{n^2},\ \mu[4,0]\to \frac{(1+n)(2+n)(3+n)}{n^3},\ \mu[4,1]\to \frac{4(1+n)(2+n)(3+n)}{n^3},\ \mu[5,0]\to \frac{(1+n)(2+n)(3+n)(4+n)}{n^4}\right\};$$

In[•]:=
```
SinfNBser[s_, 4] :=
```

$$\frac{2n}{n+1}s - \frac{4(n(1+2n))s^2}{3(1+n)^2} + \frac{2n(5+17n+14n^2)s^3}{9(1+n)^3} - \frac{8n(17+78n+117n^2+58n^3)s^4}{135(1+n)^4}$$

In[•]:=
```
FullSimplify[Series[gammaser[s, 4] /. δlist /. γlist /. substμNB, {s, 0, 4}]]
```

Out[•]=

$$1 - s + \frac{2(2+n)s^2}{3(1+n)} - \frac{4(2+n)^2 s^3}{9(1+n)^2} + \frac{2(2+n)(79+n(79+22n))s^4}{135(1+n)^3} + O[s]^5$$

7. Generalized Poisson distribution (Sect. 5.8)

Generalized Poisson distribution (Consul and Jain 1973, Tuenter 2006), where m > 0 and $0 \le \lambda < 1$. In Johnson and Kotz (2005, Univariate Discrete Distribution, 7.2 .6), this is called the Lagrangian Poisson distribution

7.1 . Basics

In[•]:=
```
GenPoi[μ_, λ_, k_] := (μ (μ + k λ) ^ (k - 1))/(k !) Exp[-μ - k λ]
```

Fix mean =1.1 and change λ; then μ = mean $(1 - \lambda)$:

In[∘]:=
```
ListLinePlot[{Table[{k, GenPoi[1.1, 0, k]}, {k, 0, 8}],
  Table[{k, GenPoi[1.1 (1 - 0.1), 0.1, k]}, {k, 0, 8}],
  Table[{k, GenPoi[1.1 (1 - 0.2), 0.2, k]}, {k, 0, 8}],
  Table[{k, GenPoi[1.1 (1 - 0.5), 0.5, k]}, {k, 0, 8}],
  Table[{k, GenPoi[1.1 (1 - 0.9) 2, 0.9, k]}, {k, 0, 8}]},
 PlotRange → All, PlotStyle → {Black, Red, Blue, Green, Pink}]
```

Out[∘]=

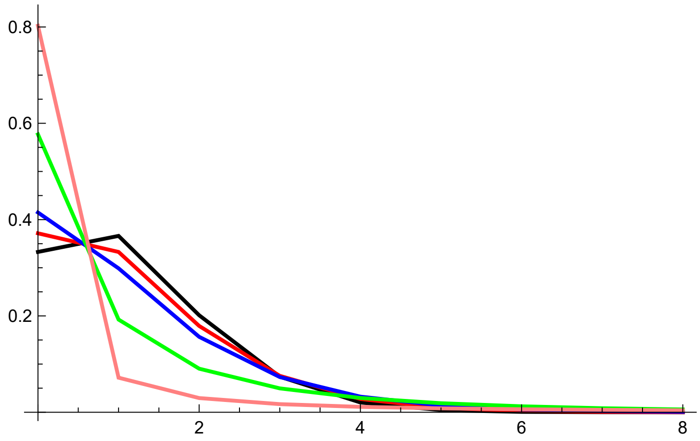

In[∘]:=
```
Table[varGP[(1 + 0.1) (1 - λ), λ], {λ, {0, 0.1, 0.2, 0.5, 0.9}}]
```

Out[∘]=
```
{1.1, 1.35802, 1.71875, 4.4, 110.}
```

7.2 . Series expansion of S^{∞}_{GP} and γ_{GP}

Fix mean m = 1 + s. Then $\mu = (1 + s)(1 - \lambda)$

The following takes a while

```
FullSimplify[
  Table[μ[k, l] → D[D[φGP[(1 + s) (1 - λ), λ][x], {x, k}], {s, l}] /. {x → 1, s → 0},
   {k, 1, 5}, {l, 0, 5 - k}], Assumptions → s > 0 && 0 ≤ λ < 1] // Flatten
```

Therefore, we define it from the output:

In[∘]:=

$$\text{subst}\mu\text{GP} = \Big\{\theta \to 2(1-\lambda)^2,\ \mu[1,0]\to 1,\ 1\to 1,\ 0\to 0,\ 0\to 0,\ 0\to 0,\ \mu[2,0]\to \frac{1}{(-1+\lambda)^2},$$
$$\mu[2,1]\to 1+\frac{1}{(-1+\lambda)^2},\ \mu[2,2]\to 2,\ \mu[2,3]\to 0,\ \mu[3,0]\to \frac{1+2\lambda}{(-1+\lambda)^4},$$
$$\mu[3,1]\to \frac{3-(-3+\lambda)(-1+\lambda)\lambda^2}{(-1+\lambda)^4},\ \mu[3,2]\to \frac{6}{(-1+\lambda)^2},\ \mu[4,0]\to \frac{1+\lambda\left(6+9\lambda-\lambda^3\right)}{(-1+\lambda)^6},$$
$$\mu[4,1]\to \frac{4+\lambda(12+\lambda(-6+\lambda(-8+\lambda(23+2(-6+\lambda)\lambda))))}{(-1+\lambda)^6},$$
$$\mu[5,0]\to \frac{1+\lambda(12+\lambda(48+\lambda(64+5\lambda(-1+(-4+\lambda)\lambda))))}{(-1+\lambda)^8}\Big\};$$

In[◦]:=
```
FullSimplify[Series[Sinfser[s, 4] /. δlist /. substμGP, {s, 0, 4}]]
```

Out[◦]=

$$2\,(-1+\lambda)^2\,s-\frac{2}{3}\left((-1+\lambda)^2\,(4+\lambda\,(-10+3\,\lambda))\right)s^2+\frac{4}{9}\,(-1+\lambda)^3\,(-7+\lambda\,(31+3\,(-7+\lambda)\,\lambda))\,s^3-\frac{2}{135}\left((-1+\lambda)^4\,(232+\lambda\,(-1552+15\,\lambda\,(116+3\,(-12+\lambda)\,\lambda)))\right)s^4+O[s]^5$$

In[◦]:=
```
Expand[(232 + λ (-1552 + 15 λ (116 + 3 (-12 + λ) λ)))]
```

Out[◦]=

$$232-1552\,\lambda+1740\,\lambda^2-540\,\lambda^3+45\,\lambda^4$$

In[◦]:=
```
SinfGPser[s_, 4] :=
```

$$2\,(1-\lambda)^2\,s-\frac{2}{3}\left((1-\lambda)^2\,(4-10\,\lambda+3\,\lambda\verb|^|2)\right)s^2+\frac{4}{9}\,(1-\lambda)^3\left(7-31\,\lambda+21\,\lambda^2-3\,\lambda^3\right)s^3-\frac{2}{135}\left((1-\lambda)^4\left(232-1552\,\lambda+1740\,\lambda^2-540\,\lambda^3+45\,\lambda^4\right)\right)s^4$$

In[◦]:=
```
Simplify[Series[gammaser[s, 4] /. δlist /. γlist /. substμGP, {s, 0, 4}]]
```

Out[◦]=

$$1-s+\frac{2}{3}\,(1+2\,\lambda)\,s^2-\frac{4}{9}\left(1+7\,\lambda+\lambda^2\right)s^3+\frac{4}{135}\left(11+162\,\lambda+93\,\lambda^2+4\,\lambda^3\right)s^4+O[s]^5$$

In[◦]:=
```
gammaGPser[s_, 4] :=
```

$$1-s+\frac{2}{3}\,(1+2\,\lambda)\,s^2-\frac{4}{9}\left(1+7\,\lambda+\lambda^2\right)s^3+\frac{4}{135}\left(11+162\,\lambda+93\,\lambda^2+4\,\lambda^3\right)s^4;$$

7.3 . Study fGP = φGP - φGPFL with the help of the series expansion

Determine the parameters π and ρ for the bounding fractional linear generating function φGPFL by series expansion

We use (3.2) -- (3.4) in the ms to calculate the fractional linear approximations

In[∘]:=
```
FullSimplify[{(1 - a2)/(1 - a1 a2), a1 (1 - a2)/(1 - a1 a2)} /. {a1 → 1 - SinfGPser[s, 4], a2 → gammaGPser[s, 4]}]
```

Out[∘]=

$$\left\{\left(s-\frac{2}{3}s^2(1+2\lambda)+\frac{4}{9}s^3(1+\lambda(7+\lambda))-\frac{4}{135}s^4(11+\lambda(162+\lambda(93+4\lambda)))\right)\Big/\left(1-\frac{1}{18225}\left(135+2s(-1+\lambda)^2\left(-135+45s(4+\lambda(-10+3\lambda))-30s^2(-1+\lambda)(-7+\lambda(31+3(-7+\lambda)\lambda))+s^3(-1+\lambda)^2(232+\lambda(-1552+15\lambda(116+3(-12+\lambda)\lambda)))\right)\right)\left(135+s\left(-135+2s\left(45+90\lambda-30s(1+\lambda(7+\lambda))+2s^2(11+\lambda(162+\lambda(93+4\lambda)))\right)\right)\right)\right),\right.$$

$$\left.\left(\left(s-\frac{2}{3}s^2(1+2\lambda)+\frac{4}{9}s^3(1+\lambda(7+\lambda))-\frac{4}{135}s^4(11+\lambda(162+\lambda(93+4\lambda)))\right)\left(1+\frac{2}{135}s(-1+\lambda)^2\left(-135+45s(4+\lambda(-10+3\lambda))-30s^2(-1+\lambda)(-7+\lambda(31+3(-7+\lambda)\lambda))+s^3(-1+\lambda)^2(232+\lambda(-1552+15\lambda(116+3(-12+\lambda)\lambda)))\right)\right)\right)\Big/\left(1-\frac{1}{18225}\left(135+2s(-1+\lambda)^2\left(-135+45s(4+\lambda(-10+3\lambda))-30s^2(-1+\lambda)(-7+\lambda(31+3(-7+\lambda)\lambda))+s^3(-1+\lambda)^2(232+\lambda(-1552+15\lambda(116+3(-12+\lambda)\lambda)))\right)\right)\left(135+s\left(-135+2s\left(45+90\lambda-30s(1+\lambda(7+\lambda))+2s^2(11+\lambda(162+\lambda(93+4\lambda)))\right)\right)\right)\right)\right\}$$

We use this result to define the substitution:

In[∘]:=
```
substprGPs4[s_, λ_] :=
  {p → (s - 2/3 s^2 (1 + 2 λ) + 4/9 s^3 (1 + λ (7 + λ)) - 4/135 s^4 (11 + λ (162 + λ (93 + 4 λ))))/
     (1 - 1/18225 (135 + 2 s (-1 + λ)^2
          (-135 + 45 s (4 + λ (-10 + 3 λ)) - 30 s^2 (-1 + λ) (-7 + λ (31 + 3 (-7 + λ) λ)) +
            s^3 (-1 + λ)^2 (232 + λ (-1552 + 15 λ (116 + 3 (-12 + λ) λ))))) (135 + s (-135 +
            2 s (45 + 90 λ - 30 s (1 + λ (7 + λ)) + 2 s^2 (11 + λ (162 + λ (93 + 4 λ))))))),
   r → ((s - 2/3 s^2 (1 + 2 λ) + 4/9 s^3 (1 + λ (7 + λ)) - 4/135 s^4 (11 + λ (162 + λ (93 + 4 λ))))
       (1 + 2/135 s (-1 + λ)^2
          (-135 + 45 s (4 + λ (-10 + 3 λ)) - 30 s^2 (-1 + λ) (-7 + λ (31 + 3 (-7 + λ) λ)) +
            s^3 (-1 + λ)^2 (232 + λ (-1552 + 15 λ (116 + 3 (-12 + λ) λ))))))/
     (1 - 1/18225 (135 + 2 s (-1 + λ)^2 (-135 + 45 s (4 + λ (-10 + 3 λ)) -
            30 s^2 (-1 + λ) (-7 + λ (31 + 3 (-7 + λ) λ)) +
            s^3 (-1 + λ)^2 (232 + λ (-1552 + 15 λ (116 + 3 (-12 + λ) λ))))) (135 + s (-135 +
            2 s (45 + 90 λ - 30 s (1 + λ (7 + λ)) + 2 s^2 (11 + λ (162 + λ (93 + 4 λ)))))))};
```

Calculate the series expansion of fGP = φGP - φGPFL

In[•]:=
```
FullSimplify[Series[φGP[(1 + s) (1 - λ), λ][x] - φFL[p, r][x] /. substprGPs4[s, λ],
  {s, 0, 2}], Assumptions → s > 0 && 0 ≤ λ < 1 && 0 ≤ x ≤ 1]
```

Out[•]=

$$\left(e^{\frac{(-1+\lambda)\,(\lambda+\text{ProductLog}[-e^{-\lambda}\,x\,\lambda])}{\lambda}} + \frac{1+x+2\,x\,(-2+\lambda)\,\lambda}{-3+x-2\,(-2+\lambda)\,\lambda}\right) + \left(-\frac{2\,(-1+x)\,(-1+\lambda)^2\,(4+\lambda\,(2+3\,\lambda)+x\,(2+\lambda\,(-14+3\,\lambda)))}{3\,(-3+x-2\,(-2+\lambda)\,\lambda)^2} + \frac{e^{\frac{(-1+\lambda)\,(\lambda+\text{ProductLog}[-e^{-\lambda}\,x\,\lambda])}{\lambda}}\,(-1+\lambda)\,(\lambda+\text{ProductLog}[-e^{-\lambda}\,x\,\lambda])}{\lambda}\right) s + \frac{1}{18}\,(-1+\lambda)^2\left(\frac{1}{(-3+x-2\,(-2+\lambda)\,\lambda)^3}\,8\,(-1+x)\,\Big(-7+3\,x^2\,\big(1+\lambda\,(-14+(-5+\lambda)^2\,\lambda)\big)+\lambda\,(-14+\lambda\,(27+\lambda\,(22+\lambda\,(-22-3\,(-2+\lambda)\,\lambda))))+x\,(10+\lambda\,(8+\lambda\,(30+\lambda\,(-160+\lambda\,(121+3\,(-10+\lambda)\,\lambda)))))\Big) + \frac{9\,e^{\frac{(-1+\lambda)\,(\lambda+\text{ProductLog}[-e^{-\lambda}\,x\,\lambda])}{\lambda}}\,(\lambda+\text{ProductLog}[-e^{-\lambda}\,x\,\lambda])^2}{\lambda^2}\right) s^2 + O[s]^3$$

The above is slow, there we use the result to define

In[•]:=

$$\text{fGPser2}[s\_,\lambda\_][x\_] := \left(e^{\frac{(-1+\lambda)\,(\lambda+\text{ProductLog}[-e^{-\lambda}\,x\,\lambda])}{\lambda}} + \frac{1+x+2\,x\,(-2+\lambda)\,\lambda}{-3+x-2\,(-2+\lambda)\,\lambda}\right) + \left(-\frac{2\,(-1+x)\,(-1+\lambda)^2\,(4+\lambda\,(2+3\,\lambda)+x\,(2+\lambda\,(-14+3\,\lambda)))}{3\,(-3+x-2\,(-2+\lambda)\,\lambda)^2} + \frac{e^{\frac{(-1+\lambda)\,(\lambda+\text{ProductLog}[-e^{-\lambda}\,x\,\lambda])}{\lambda}}\,(-1+\lambda)\,(\lambda+\text{ProductLog}[-e^{-\lambda}\,x\,\lambda])}{\lambda}\right) s + \frac{1}{18}\,(-1+\lambda)^2\left(\frac{1}{(-3+x-2\,(-2+\lambda)\,\lambda)^3}\,8\,(-1+x)\,\Big(-7+3\,x^2\,\big(1+\lambda\,(-14+(-5+\lambda)^2\,\lambda)\big)+\lambda\,(-14+\lambda\,(27+\lambda\,(22+\lambda\,(-22-3\,(-2+\lambda)\,\lambda))))+x\,(10+\lambda\,(8+\lambda\,(30+\lambda\,(-160+\lambda\,(121+3\,(-10+\lambda)\,\lambda)))))\Big) + \frac{9\,e^{\frac{(-1+\lambda)\,(\lambda+\text{ProductLog}[-e^{-\lambda}\,x\,\lambda])}{\lambda}}\,(\lambda+\text{ProductLog}[-e^{-\lambda}\,x\,\lambda])^2}{\lambda^2}\right) s^2$$

Analysis of fGPser2 (eqs. (5.35) - (5.39))

The following gives (5.35):

In[ ]:= `Simplify[Series[fGPser2[s, λ][0], {s, 0, 2}]]`

Out[ ]=

$$\left(e^{-1+\lambda}+\frac{1}{-3-2\,(-2+\lambda)\,\lambda}\right)+\left(e^{-1+\lambda}\,(-1+\lambda)+\frac{2\,(-1+\lambda)^2\,(4+\lambda\,(2+3\,\lambda))}{3\left(3-4\,\lambda+2\,\lambda^2\right)^2}\right)s+\frac{1}{18}\,(-1+\lambda)^2\left(9\,e^{-1+\lambda}-\frac{8\,(-7+\lambda\,(-14+\lambda\,(27+\lambda\,(22+\lambda\,(-22-3\,(-2+\lambda)\,\lambda)))))}{(-3-2\,(-2+\lambda)\,\lambda)^3}\right)s^2+O[s]^3$$

Now show (5.36):

In[ ]:=

$$\text{NSolve}\left[e^{-1+\lambda}+\frac{1}{-3+4\,\lambda-2\,\lambda^2}==0,\ \lambda,\ \text{Reals}\right]$$

Out[ ]=

$$\{\{\lambda\to-1.84267\},\ \{\lambda\to0.25915\},\ \{\lambda\to1.\}\}$$

In[ ]:=

$$\text{Simplify}\left[\text{Series}\left[\left(e^{-1+\lambda}+\frac{1}{-3-2\,(-2+\lambda)\,\lambda}\right)+\left(e^{-1+\lambda}\,(-1+\lambda)+\frac{2\,(-1+\lambda)^2\,(4+\lambda\,(2+3\,\lambda))}{3\left(3-4\,\lambda+2\,\lambda^2\right)^2}\right)s\ /.\ \lambda\to\lambda0+\lambda01\,s,\ \{s,0,1\}\right]\right]$$

Out[ ]=

$$\left(e^{-1+\lambda0}+\frac{1}{-3-2\,(-2+\lambda0)\,\lambda0}\right)+\left(e^{-1+\lambda0}\,(-1+\lambda0)+\frac{2\,(-1+\lambda0)^2\,(4+\lambda0\,(2+3\,\lambda0))}{3\left(3-4\,\lambda0+2\,\lambda0^2\right)^2}+e^{-1+\lambda0}\,\lambda01+\frac{4\,(-1+\lambda0)\,\lambda01}{\left(3-4\,\lambda0+2\,\lambda0^2\right)^2}\right)s+O[s]^2$$

In[ ]:=

$$\text{NSolve}\left[\left(\left(e^{-1+\lambda0}\,(-1+\lambda0)+\frac{2\,(-1+\lambda0)^2\,(4+\lambda0\,(2+3\,\lambda0))}{3\left(3-4\,\lambda0+2\,\lambda0^2\right)^2}+e^{-1+\lambda0}\,\lambda01+\frac{4\,(-1+\lambda0)\,\lambda01}{\left(3-4\,\lambda0+2\,\lambda0^2\right)^2}\right)/.\ \lambda0\to0.2591495702201173\text{`}\right)==0,\ \lambda01\right]$$

Out[ ]=

$$\{\{\lambda01\to0.199726\}\}$$

Show (5.37) :

In[ ]:= `Simplify[Series[(1 + s) gammaGPser[s, 4], {s, 0, 3}]]`

Out[ ]=

$$1+\frac{1}{3}\,(-1+4\,\lambda)\,s^2-\frac{2}{9}\left(-1+8\,\lambda+2\,\lambda^2\right)s^3+O[s]^4$$

In[ ]:=

$$\text{Simplify}\left[\text{Solve}\left[\frac{1}{3}\,(-1+4\,\lambda)-\frac{2}{9}\left(-1+8\,\lambda+2\,\lambda^2\right)s==0,\ \lambda\right]\right]$$

Out[ ]=

$$\left\{\left\{\lambda\to\frac{3-4\,s-3\,\sqrt{1-3\,s+2\,s^2}}{2\,s}\right\},\ \left\{\lambda\to\frac{3-4\,s+3\,\sqrt{1-3\,s+2\,s^2}}{2\,s}\right\}\right\}$$

In[ ]:=

$$\text{Simplify}\left[\text{Series}\left[\frac{3-4\,s-3\,\sqrt{1-3\,s+2\,s^2}}{2\,s},\ \{s,0,1\}\right]\right]$$

Out[ ]=

$$\frac{1}{4}+\frac{3\,s}{16}+O[s]^2$$

Show (5.38) and (5.39) :

In[•]:=
```
FullSimplify[Series[D[fGPser2[s, λ][x], {x, 2}] /. x → 1 - SinfGPser[s, 4], {s, 0, 2}],
  Assumptions → s > 0 && 0 ≤ λ < 1]
```

Out[•]=

$$\frac{(1-4\lambda)\,s}{3\,(-1+\lambda)^2}-\frac{(1+\lambda\,(4+\lambda\,(-74+3\,\lambda\,(4+\lambda))))\,s^2}{9\,(-1+\lambda)^2}+O[s]^3$$

In[•]:=
```
Simplify[Series[(1 - 4 λ)/3 - (1 + λ (4 + λ (-74 + 3 λ (4 + λ)))) s/9, {λ, 1/4, 1}]] // Normal
```

Out[•]=

$$\frac{69\,s}{256}+\left(-\frac{4}{3}+\frac{163\,s}{48}\right)\left(-\frac{1}{4}+\lambda\right)$$

In[•]:=
```
Solve[% == 0, λ]
```

Out[•]=

$$\left\{\left\{\lambda\to\frac{-256+445\,s}{16\,(-64+163\,s)}\right\}\right\}$$

In[•]:=
```
N[Normal[Series[(-256 + 445 s)/(16 (-64 + 163 s)), {s, 0, 1}]]]
```

Out[•]=

0.25 + 0.202148 s

7.4 . The numerically exact version of fGP and Figure 5.1

We define fGP such that it is efficient for calculations required for graphics and tables. We use (3.3) and (3.4) in the ms:

In[•]:=
```
fGP[μ_, λ_][x_] := (a1 = PinfGP[μ, λ];
  a2 = gammaGP[μ, λ];
  φGP[μ, λ][x] - φFL[(1 - a2)/(1 - a1 a2), a1 (1 - a2)/(1 - a1 a2)][x])
```

In[•]:=
```
plotfGPfig =
  Plot[{fGP[(1 + 0.3) (1 - 0.30), 0.30][x], fGP[(1 + 0.3) (1 - 0.3035), 0.3035][x],
    fGP[(1 + 0.3) (1 - 0.31), 0.31][x], fGP[(1 + 0.3) (1 - 0.314), 0.314][x],
    fGP[(1 + 0.3) (1 - 0.3145), 0.3145][x]}, {x, 0, 1}, AxesLabel → {"x", "f_GP(x)"},
   LabelStyle → {FontFamily → "Helvetica", FontSize → 13, Black},
   PlotStyle → {Blue, Red, Black, Darker[Green], Orange},
   PlotRange → {-0.00009, 0.000052}, ImageSize → 450];
```

In[•]:=
```
plotlegend = LineLegend[{Blue, Red, Black, Darker[Green], Orange},
   {"0.3000", "0.3035", "0.3100", "0.3140", "0.3145"}, LegendLabel → " λ",
   LabelStyle → Directive[FontFamily → "Helvetica", FontSize → 12], LegendFunction →
    (Framed[#, RoundingRadius → 4, FrameStyle → LightGray, ContentPadding → False] &)];
```

In[•]:=
```
Show[Legended[plotfGPfig, Placed[plotlegend, {1.05, 0.5}]], ImageSize → 600]
```

Out[•]=

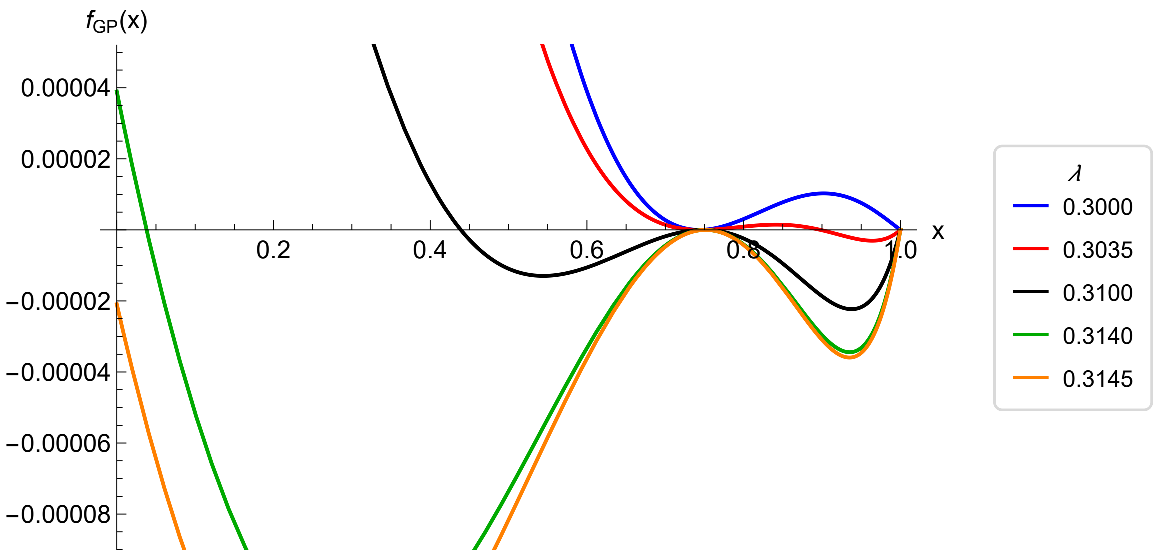

In[•]:=
```
plotfGPfigall =
  Plot[{fGP[(1 + 0.3) (1 - 0.30), 0.30][x], fGP[(1 + 0.3) (1 - 0.3035), 0.3035][x],
    fGP[(1 + 0.3) (1 - 0.31), 0.31][x], fGP[(1 + 0.3) (1 - 0.314), 0.314][x],
    fGP[(1 + 0.3) (1 - 0.3145), 0.3145][x]}, {x, 0, 1}, AxesLabel → {"x", "fGP(x)"},
   LabelStyle → {FontFamily → "Helvetica", FontSize → 13, Black}, PlotStyle →
    {Blue, Red, Black, Darker[Green], Orange}, PlotRange → All, ImageSize → 450];
```

In[•]:=
```
plotfGPfigall
```

Out[•]=

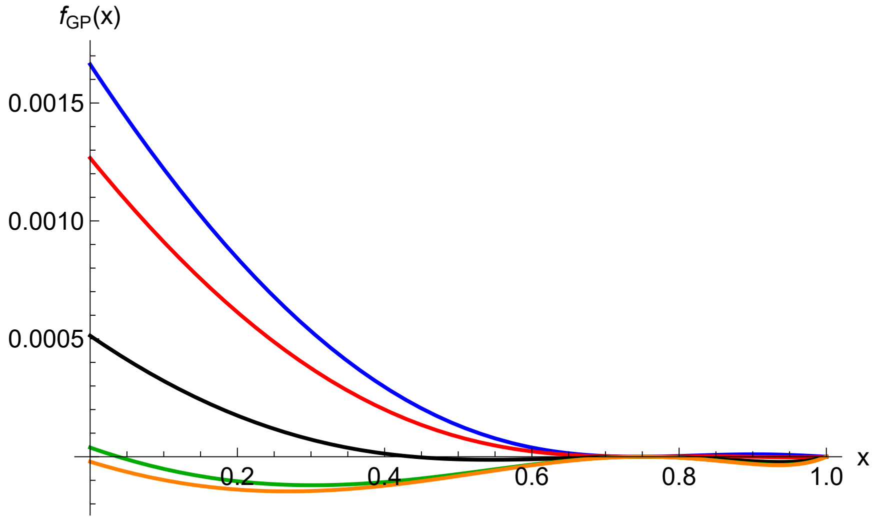

8. Convergence time $T_\varphi(\epsilon)$ and graphs of relative errors of survival probabilities $S_\varphi^{(n)}$ (Sects. 6.1, 6.2)

8.1 Calculate $T_{app}(\epsilon)$ and $T_{ser}(\epsilon)$

Definitions

The following is the approximation (6.1) obtained from the bound by the fractional linear distribution:

In[∘]:=
```
TepFLapp[Pinf_, gam_, ϵ_] := Ceiling[Log[(1 + 1 / ϵ) Pinf] / (-Log[gam])]
```

Show (6.2):

In[∘]:=
```
gammaser[s, 3]
```

Out[∘]=

$$-s + s^2 \gamma[2] - s^3 \gamma[3]$$

In[∘]:=
```
Simplify[Series[Log[(1 + 1 / ϵ) Pinf] / (-Log[gam]) /. Pinf → 1 - Sinfser[s, 3] /. gam → gammaser[s, 3] /.
   substderiv, {s, 0, 1}], Assumptions → s > 0]
```

Out[∘]=

$$\frac{\mathrm{Log}\left[1+\frac{1}{\epsilon}\right]}{s} + \left(-\theta + \mathrm{Log}\left[1+\frac{1}{\epsilon}\right]\left(-\frac{1}{2}+\gamma[2]\right)\right) + \left(\mathrm{Log}\left[1+\frac{1}{\epsilon}\right]\left(-\frac{1}{12}+\gamma[2]^2-\gamma[3]\right)+\frac{1}{2}\left(\theta-\theta^2-2\,\theta\,\gamma[2]+2\,\delta[2]\right)\right) s + O[s]^2$$

In[∘]:=
```
TepSer[s_, ϵ_] := Ceiling[(1/s - 1/2 + γ[2]) Log[1 + 1/ϵ] - θ]
```

Calculate values in Table 6.1

The following computes TepFLapp for the generating functions introduced above

In[∘]:=
```
TepFLPoi[m_, ϵ_] := TepFLapp[PinfPoi[m], gammaPoi[m], ϵ] // Ceiling;
TepFLGP[μ_, λ_, ϵ_] := TepFLapp[PinfGP[μ, λ], gammaGP[μ, λ], ϵ] // Ceiling;
TepFLBin[n_, p_, ϵ_] := TepFLapp[PinfBin[n, p], gammaBin[n, p], ϵ] // Ceiling;
TepFLNB[n_, p_, ϵ_] := TepFLapp[PinfNB[n, p], gammaNB[n, p], ϵ] // Ceiling;
TepFLF3[p0_, p2_, p3_, ϵ_] :=
  TepFLapp[PinfF3[p0, p2, p3], gammaF3[p0, p2, p3], ϵ] // Ceiling;
TepFLFL[p_, r_, ϵ_] := TepFLapp[PinfFL[p, r], gammaFL[p, r], ϵ] // Ceiling;
```

The following yields the numerically exact value by iteration of the generating function:

In[]:=
```
TepPoi[m_, ϵ_] :=
  (NestWhileList[φPoi[m], 0, 1 - #1 > (1 + ϵ) (1 - PinfPoi[m]) &] // Length) - 1;
TepListPoi[m_, ϵ_] := NestWhileList[φPoi[m], 0, 1 - #1 > (1 + ϵ) (1 - PinfPoi[m]) &];
TepGP[μ_, λ_, ϵ_] :=
  (NestWhileList[φGP[μ, λ], 0, 1 - #1 > (1 + ϵ) (1 - PinfGP[μ, λ]) &] // Length) - 1;
TepBin[n_, p_, ϵ_] :=
  (NestWhileList[φBin[n, p], 0, 1 - #1 > (1 + ϵ) (1 - PinfBin[n, p]) &] // Length) - 1;
TepNB[n_, p_, ϵ_] :=
  (NestWhileList[φNB[n, p], 0, 1 - #1 > (1 + ϵ) (1 - PinfNB[n, p]) &] // Length) - 1;
TepF3[p0_, p2_, p3_, ϵ_] := (NestWhileList[φF3[p0, p2, p3],
     0, 1 - #1 > (1 + ϵ) (1 - PinfF3[p0, p2, p3]) &] // Length) - 1;
TepFL[p_, r_, ϵ_] :=
  (NestWhileList[φFL[p, r], 0, 1 - #1 > (1 + ϵ) (1 - PinfFL[p, r]) &] // Length) - 1;
```

Some examples :

s = 0.01

In[]:=
```
Table[{"ϵ" → ϵ, Table[TepGP[(1 + 0.01) (1 - λ), λ, ϵ], {λ, {0, 0.1, 0.259, 0.5, 0.9}}]},
 {ϵ, {0.01}}]
```
Out[]=
```
{{ϵ → 0.01, {459, 461, 463, 467, 474}}}
```

In[]:=
```
Table[{"ϵ" → ϵ, Table[TepFLGP[(1 + 0.01) (1 - λ), λ, ϵ], {λ, {0, 0.1, 0.259, 0.5, 0.9}}]},
 {ϵ, {0.01}}]
```
Out[]=
```
{{ϵ → 0.01, {461, 462, 463, 465, 468}}}
```

In[]:=
```
Table[
 {"ϵ" → ϵ, Table[TepSer[0.01, ϵ] /. γlist /. substμGP, {λ, {0, 0.1, 0.259, 0.5, 0.9}}]},
 {ϵ, {0.01}}]
```
Out[]=
```
{{ϵ → 0.01, {461, 462, 463, 465, 468}}}
```

s = 0.1

In[]:=
```
Table[{"ϵ" → ϵ, Table[TepGP[(1 + 0.1) (1 - λ), λ, ϵ], {λ, {0, 0.1, 0.276, 0.5, 0.9}}]},
 {ϵ, {0.1, 0.01, 0.0001}}]
```
Out[]=
```
{{ϵ → 0.1, {22, 23, 25, 27, 31}},
 {ϵ → 0.01, {45, 46, 48, 51, 56}}, {ϵ → 0.0001, {91, 93, 96, 100, 108}}}
```

In[]:=
```
Table[{"ϵ" → ϵ, Table[TepFLGP[(1 + 0.1) (1 - λ), λ, ϵ], {λ, {0, 0.1, 0.276, 0.5, 0.9}}]},
 {ϵ, {0.1, 0.01, 0.0001}}]
```
Out[]=
```
{{ϵ → 0.1, {23, 24, 25, 26, 28}},
 {ϵ → 0.01, {45, 46, 48, 50, 53}}, {ϵ → 0.0001, {92, 94, 96, 99, 105}}}
```

In[]:=
```
Table[
 {"ϵ" → ϵ, Table[TepSer[0.1, ϵ] /. γlist /. substμGP, {λ, {0, 0.1, 0.276, 0.5, 0.9}}]},
 {ϵ, {0.1, 0.01, 0.0001}}]
```
Out[]=
```
{{ϵ → 0.1, {23, 24, 25, 26, 28}},
 {ϵ → 0.01, {45, 46, 48, 50, 53}}, {ϵ → 0.0001, {92, 94, 96, 100, 105}}}
```

In[]:=
```
Table[{"ϵ" → ϵ, TepNB[5, 5/(5 + 1.1), ϵ]}, {ϵ, {0.1, 0.01, 0.0001}}]
```
Out[]=
```
{{ϵ → 0.1, 23}, {ϵ → 0.01, 46}, {ϵ → 0.0001, 93}}
```
In[]:=
```
Table[{"ϵ" → ϵ, TepFLNB[5, 5/(5 + 1.1), ϵ]}, {ϵ, {0.1, 0.01, 0.0001}}]
```
Out[]=
```
{{ϵ → 0.1, 23}, {ϵ → 0.01, 46}, {ϵ → 0.0001, 93}}
```
In[]:=
```
Table[{"ϵ" → ϵ, TepSer[0.1, ϵ] /. γlist /. substμNB /. n → 5}, {ϵ, {0.1, 0.01, 0.0001}}]
```
Out[]=
```
{{ϵ → 0.1, 23}, {ϵ → 0.01, 46}, {ϵ → 0.0001, 93}}
```
s = 0.3

In[]:=
```
Table[{"ϵ" → ϵ, Table[TepGP[(1 + 0.3) (1 - λ), λ, ϵ], {λ, {0, 0.2, 0.312, 0.5, 0.9}}]},
 {ϵ, {0.01}}]
```
Out[]=
```
{{ϵ → 0.01, {14, 16, 17, 19, 24}}}
```
In[]:=
```
Table[{"ϵ" → ϵ, Table[TepFLGP[(1 + 0.3) (1 - λ), λ, ϵ], {λ, {0, 0.2, 0.312, 0.5, 0.9}}]},
 {ϵ, {0.01}}]
```
Out[]=
```
{{ϵ → 0.01, {15, 16, 17, 19, 22}}}
```
In[]:=
```
Table[
 {"ϵ" → ϵ, Table[TepSer[0.3, ϵ] /. γlist /. substμGP, {λ, {0, 0.2, 0.312, 0.5, 0.9}}]},
 {ϵ, {0.01}}]
```
Out[]=
```
{{ϵ → 0.01, {15, 17, 18, 19, 22}}}
```

8.2 Relative errors of survival probabilities $S_{\varphi}^{(n)}$ and Figure 6.1

Define iterates 1,2, 3,...,t of generating functions

In[]:=
```
φitPoi[m_, t_][x_] := Delete[NestList[φPoi[m], x, t], 1];
φitFL[p_, r_, t_][x_] := Delete[NestList[φFL[p, r], x, t], 1];
φitBin[n_, p_, t_][x_] := Delete[NestList[φBin[n, p], x, t], 1];
φitNB[n_, p_, t_][x_] := Delete[NestList[φNB[n, p], x, t], 1];
φitF3[p0_, p2_, p3_, t_][x_] := Delete[NestList[φF3[p0, p2, p3], x, t], 1];
φitGP[μ_, λ_, t_][x_] := Delete[NestList[φGP[μ, λ], x, t], 1];
```
In[]:=
```
φitPoi[1.1, 5][0.3]
```
Out[]=
```
{0.463013, 0.553947, 0.612223, 0.652754, 0.682515}
```
The following are the iterates of the fractional linear approximation to the given generating function

In[•]:=
```
φitPoiFL[m_, t_][x_] := (φitFL[p, r, t] /. substprPoi[m])[x];
φitBinFL[n_, pp_, t_][x_] := (φitFL[p, r, t] /. substprBin[n, pp])[x];
φitNBFL[n_, pp_, t_][x_] := (φitFL[p, r, t] /. substprNB[n, pp])[x];
φitF3FL[p0_, p2_, p3_, t_][x_] := (φitFL[p, r, t] /. substprF3[p0, p2, p3])[x];
φitGPFL[μ_, λ_, t_][x_] := (φitFL[p, r, t] /. substprGP[μ, λ])[x];
```

The following gives yields extinction probabilities from generation 1 -- 15 :

In[•]:=
```
φitBin[10, 0.12, 15][0]
```

Out[•]=
```
{0.278501, 0.404302, 0.476316, 0.52255, 0.554323, 0.577154, 0.59408,
 0.606915, 0.616812, 0.624544, 0.630645, 0.635497, 0.639379, 0.6425, 0.64502}
```

Plot relative errors of extinction probabilities, (φitGP[pars][0] - φitGPFL[pars][0])/(1 - φitGP[pars][0]), for the generalized Poisson

Generalized Poisson for s = 0.1, λ given in the legend

In[•]:=
```
plotSnGP = Show[{ListPlot[(φitGP[1.1, 0, 40][0] - φitGPFL[1.1, 0, 40][0]) /
      (1 - φitGP[1.1, 0, 40][0]), PlotStyle → Red, PlotRange → All],
    ListPlot[(φitGP[1.1 (1 - 0.1), 0.1, 40][0] - φitGPFL[1.1 (1 - 0.1), 0.1, 40][0]) /
      (1 - φitGP[1.1 (1 - 0.1), 0.1, 40][0]), PlotStyle → Blue, PlotRange → All],
    ListPlot[
     (φitGP[1.1 (1 - 0.276), 0.276, 40][0] - φitGPFL[1.1 (1 - 0.276), 0.276, 40][0]) /
      (1 - φitGP[1.1 (1 - 0.276), 0.276, 40][0]), PlotStyle → Orange, PlotRange → All],
    ListPlot[(φitGP[1.1 (1 - 0.4), 0.4, 40][0] - φitGPFL[1.1 (1 - 0.4), 0.4, 40][0]) /
      (1 - φitGP[1.1 (1 - 0.4), 0.4, 40][0]), PlotStyle → Black, PlotRange → All],
    ListPlot[(φitGP[1.1 (1 - 0.5), 0.5, 40][0] - φitGPFL[1.1 (1 - 0.5), 0.5, 40][0]) /
      (1 - φitGP[1.1 (1 - 0.5), 0.5, 40][0]), PlotStyle → Darker[Green], PlotRange → All]},
   PlotRange → All, AxesOrigin → {1, 0}, ImageSize → 450];
```

In[•]:=
```
plotlegendSnGP = PointLegend[{Red, Blue, Orange, Black, Darker[Green]},
   {"0", "0.1", "0.276", "0.4", "0.5"}, LegendLabel → "λ  ",
   LabelStyle → Directive[FontFamily → "Helvetica", FontSize → 12], LegendFunction →
    (Framed[#, RoundingRadius → 4, FrameStyle → LightGray, ContentPadding → False] &)];
```

In[∘]:=
```
Show[Legended[plotSnGP, Placed[plotlegendSnGP, {0.85, 0.33}]]]
```

Out[∘]=

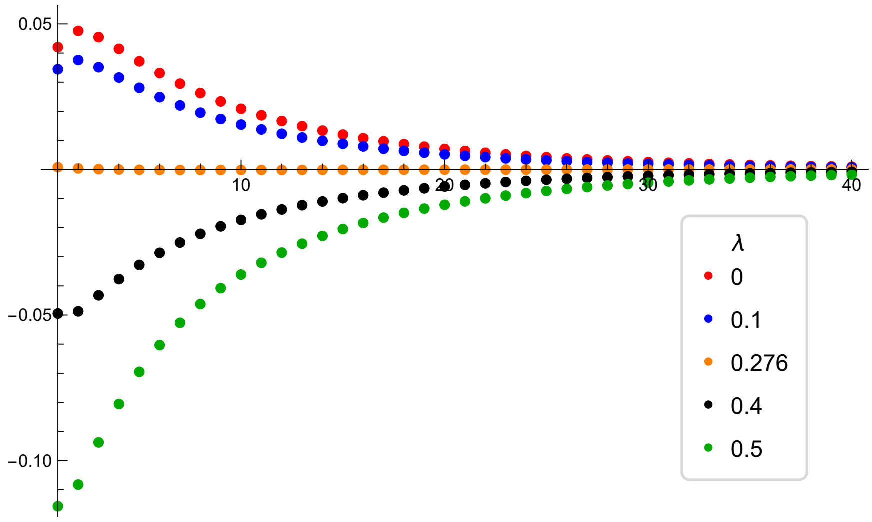

8.3 Series approximation of $S_{GP}^{(n)}$

Approximate $S_{GP}^{(n)}$ by using SinfGPser and gammaGPser

We define approximations up to s^3 :

In[∘]:=
```
SinfGPser3[s_, λ_] :=
  2 (1 - λ)^2 s - 2/3 ((1 - λ)^2 (4 - 10 λ + 3 λ^2)) s^2 + 4/9 (1 - λ)^3 (7 - 31 λ + 21 λ^2 - 3 λ^3) s^3;
gammaGPser3[s_, λ_] := 1 - s + 2/3 (1 + 2 λ) s^2 - 4/9 (1 + 7 λ + λ^2) s^3
```

The following is the approx to Sn using the approx :

In[∘]:=
```
SnGPser3[s_, λ_, n_] := SinfGPser3[s, λ] / (1 - gammaGPser3[s, λ]^n (1 - SinfGPser3[s, λ]));
```

The following shows the relative errors of this approx (for the same parameters as above):

In[ ]:=
```
plotSnGPser3 =
 Show[{ListPlot[(φitGP[1.1, 0, 40][0] - (1 - Table[SnGPser3[0.1, 0, n], {n, 1, 40}])) /
     (1 - φitGP[1.1, 0, 40][0]), PlotStyle → Red, PlotRange → All], ListPlot[
    (φitGP[1.1 (1 - 0.1), 0.1, 40][0] - (1 - Table[SnGPser3[0.1, 0.1, n], {n, 1, 40}])) /
     (1 - φitGP[1.1 (1 - 0.1), 0.1, 40][0]), PlotStyle → Blue, PlotRange → All],
   ListPlot[(φitGP[1.1 (1 - 0.276), 0.276, 40][0] -
       (1 - Table[SnGPser3[0.1, 0.276, n], {n, 1, 40}])) /
     (1 - φitGP[1.1 (1 - 0.276), 0.276, 40][0]),
    PlotStyle → Orange, PlotRange → All], ListPlot[
    (φitGP[1.1 (1 - 0.4), 0.4, 40][0] - (1 - Table[SnGPser3[0.1, 0.4, n], {n, 1, 40}])) /
     (1 - φitGP[1.1 (1 - 0.4), 0.4, 40][0]), PlotStyle → Black, PlotRange → All], ListPlot[
    (φitGP[1.1 (1 - 0.5), 0.5, 40][0] - (1 - Table[SnGPser3[0.1, 0.5, n], {n, 1, 40}])) /
     (1 - φitGP[1.1 (1 - 0.5), 0.5, 40][0]), PlotStyle → Darker[Green], PlotRange → All]},
  PlotRange → All, AxesOrigin → {1, 0}, ImageSize → 450]
```

Out[ ]=
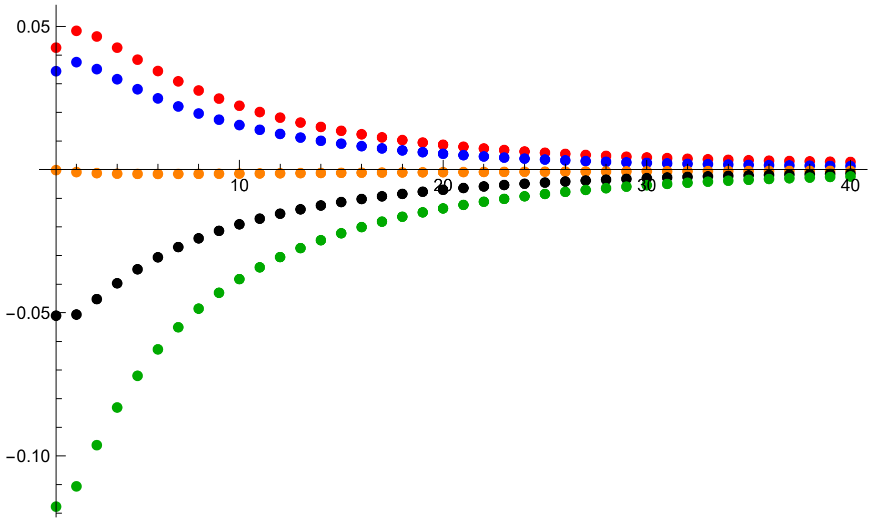

Because on this scale of resolution there is little visible difference to the above figure, below I show the relative errors of the precisely determined fractional linear approx and of the approx obtained by series expansion to order s^3 of Sinf. This is shown on a longer time scale. It illustrates the fact that the series approximation of Sinf has an error of O(s^3).

In[∘]:=
```
Show[{ListPlot[(φitGPFL[1.1, 0, 100][0] - (1 - Table[SnGPser3[0.1, 0, n], {n, 1, 100}])) /
    (1 - φitGPFL[1.1, 0, 100][0]), PlotStyle → Red, PlotRange → All], ListPlot[
   (φitGPFL[1.1 (1 - 0.1), 0.1, 100][0] - (1 - Table[SnGPser3[0.1, 0.1, n], {n, 1, 100}])) /
    (1 - φitGPFL[1.1 (1 - 0.1), 0.1, 100][0]), PlotStyle → Blue, PlotRange → All],
  ListPlot[(φitGPFL[1.1 (1 - 0.276), 0.276, 100][0] -
      (1 - Table[SnGPser3[0.1, 0.276, n], {n, 1, 100}])) /
    (1 - φitGPFL[1.1 (1 - 0.276), 0.276, 100][0]),
   PlotStyle → Orange, PlotRange → All], ListPlot[
   (φitGPFL[1.1 (1 - 0.4), 0.4, 100][0] - (1 - Table[SnGPser3[0.1, 0.4, n], {n, 1, 100}])) /
    (1 - φitGPFL[1.1 (1 - 0.4), 0.4, 100][0]),
   PlotStyle → Black, PlotRange → All], ListPlot[
   (φitGPFL[1.1 (1 - 0.5), 0.5, 100][0] - (1 - Table[SnGPser3[0.1, 0.5, n], {n, 1, 100}])) /
    (1 - φitGPFL[1.1 (1 - 0.5), 0.5, 100][0]),
   PlotStyle → Darker[Green], PlotRange → All]},
 PlotRange → All, AxesOrigin → {1, 0}, ImageSize → 450]
```

Out[∘]=

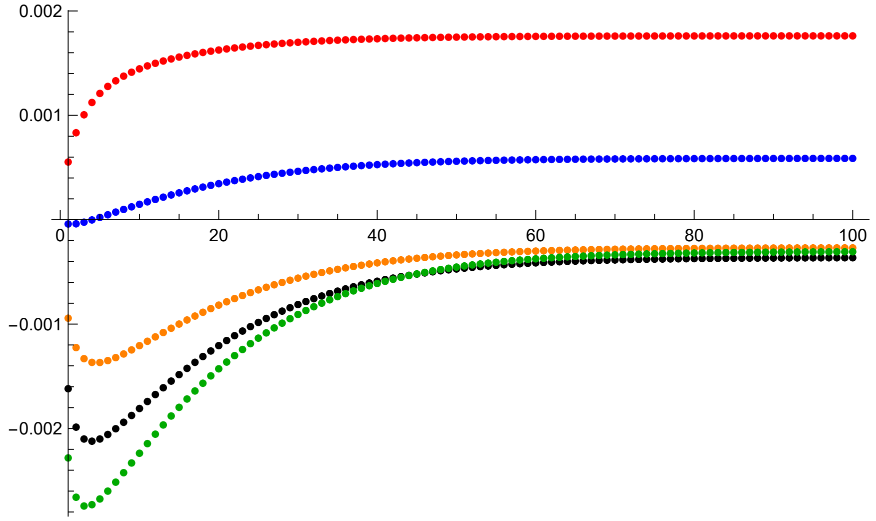

Supplement: Supplementary file 1 — (pdf 903 KB) [file 285_2026_2349_MOESM1_ESM.pdf]
